# Supplementary material for: Effect of aberrant fructose metabolism following SARS-CoV-2 infection on colorectal cancer patients’ poor prognosis
Source: PLoS Comput Biol. 2024 Sep 27;20(9):e1012412. doi: 10.1371/journal.pcbi.1012412 (PMC11463760; doi:10.1371/journal.pcbi.1012412)
Supplement: S1 Table — Cox univariate regression analysis was used to screen 333 candidate genes for prognostic associated genes. (PDF) [file pcbi.1012412.s001.pdf]

Table S1

gene	HR	z	pvalue	lower	upper
DLAT	0.710999462	-3.779482339	0.000157155	0.595732972	0.8485685
PMM2	0.670847965	-3.692514736	0.000222047	0.542746779	0.829184086
TIMP1	1.423551734	3.498708447	0.000467517	1.168030289	1.734971737
FDFT1	0.722140084	-3.358560417	0.000783496	0.597195874	0.873224888
CTNNB1	0.754347118	-3.018853068	0.002537336	0.628181916	0.90585157
AOC3	1.313686551	2.998538176	0.002712782	1.099109152	1.570155569
CLU	1.286604789	2.905767217	0.003663539	1.085484176	1.524989419
FTCD	1.105849777	2.628056308	0.00858743	1.025907558	1.192021366
PFKFB1	1.153612816	2.627419111	0.008603529	1.036968108	1.283378455
MAPK1	0.823847808	-2.547071437	0.010863119	0.709727277	0.956318339
ALDH3B1	1.275516517	2.485516505	0.012936359	1.052802482	1.545344368
SLC25A24	0.80272543	-2.365903759	0.017986119	0.669126689	0.962998676
UQCRC2	0.804865608	-2.356766942	0.018434813	0.671923548	0.964110648
GNMT	1.122755374	2.272877499	0.023033563	1.016068292	1.240644591
HSPA9	0.80444463	-2.257453132	0.023979775	0.665956885	0.97173132
PTPRD	0.800334413	-2.254445978	0.024168126	0.659444011	0.971326089
GATM	1.197160827	2.232215289	0.025600737	1.0221905	1.402081165
SERPINE1	1.219225293	2.228353932	0.025856923	1.024160974	1.45144206
IGF1	1.202600594	2.223755196	0.026164919	1.022125774	1.414941513
IL7	0.796090744	-2.197594324	0.027978028	0.649582426	0.975642885
LCAT	1.189459003	2.182946966	0.029039716	1.017880454	1.38995961
COG2	0.811006807	-2.180361109	0.029230705	0.671807832	0.979047892
STAT3	0.819194117	-2.127009271	0.033419313	0.681672604	0.984459398
CLEC4A	0.694902519	-2.123262046	0.033731899	0.49659833	0.972394551
GPI	0.819556045	-2.078221297	0.037688981	0.679320877	0.98874057
AASS	1.212171664	2.048351913	0.04052553	1.008337352	1.457210863
SDHB	0.829373266	-2.011671922	0.044254532	0.691175779	0.995202719
HARS2	0.826649905	-2.001422009	0.045346931	0.686050151	0.996064304
DDAH1	0.821645281	-1.996474443	0.045882305	0.677530635	0.996413937
PLIN1	1.176309899	1.993451205	0.046212062	1.002731522	1.379935654
GNAO1	1.180577842	1.952362845	0.050895141	0.999353905	1.394665128
UBE2Q1	1.214873823	1.951215167	0.051031456	0.999127657	1.477207038
ACLY	0.825427534	-1.931807452	0.053383276	0.679428061	1.002800226
INSR	1.207111014	1.899090481	0.05755258	0.993984638	1.465935131
CS	0.8397969	-1.867946784	0.061769489	0.699219082	1.008637853
PYGL	1.195578219	1.850225727	0.064281022	0.989461252	1.44463189
ADSL	0.811652223	-1.838472093	0.06599287	0.649756831	1.013885963
CDKN1B	0.841248413	-1.825257772	0.067962139	0.698727499	1.012839616
LMNA	1.211100123	1.815018854	0.069521012	0.984821102	1.489370512
PRKACA	1.24491712	1.812754782	0.069869651	0.982367276	1.577636668
SRSF3	0.84720107	-1.795835328	0.07252071	0.706954374	1.015270122
FCGR1A	1.170382797	1.795310103	0.072604306	0.985674265	1.389704431
RPL31	1.203976369	1.785485611	0.074182583	0.982023725	1.476093766
AGXT	1.095340264	1.772608718	0.076293566	0.990421083	1.211373943
LBR	0.849283181	-1.769304629	0.076843051	0.708695676	1.017759731
ACACB	1.186860199	1.763067553	0.077889097	0.981050088	1.435846293
XDH	0.841786218	-1.742849067	0.081359983	0.693562589	1.021687224
ATP1A1	0.852819009	-1.740331362	0.08180085	0.712832955	1.020295507
AKR1C2	1.151076512	1.705426407	0.088114927	0.979219612	1.353094975
MAPK14	0.850201344	-1.705121832	0.088171705	0.705521288	1.024550695
CYTH2	1.159620246	1.696981452	0.089700167	0.977311327	1.375937306
DBN1	1.166994146	1.659429353	0.097029306	0.972418871	1.400502783
OLR1	1.171788452	1.642301238	0.100527591	0.969801417	1.41584468
SERPINA1	0.847538892	-1.626244009	0.103897758	0.694347667	1.034528102
TTR	1.098794275	1.617138871	0.105848308	0.980225365	1.231705384
MYC	0.85288597	-1.606555016	0.108152017	0.702391713	1.035625085
PCCA	0.848133184	-1.587168723	0.112474436	0.692031241	1.039447145
SLC26A2	0.855396163	-1.553349646	0.120339626	0.702389954	1.041732718
SLC2A9	0.861210637	-1.548871511	0.121412611	0.712846269	1.040454013
PDZK1	1.142264311	1.536235508	0.12448063	0.963976982	1.353525843
EZH2	0.868320592	-1.52103183	0.12825185	0.723877029	1.041586652
ACACA	0.854823208	-1.511309423	0.130709634	0.697475701	1.047667633
ANAPC1	0.856822558	-1.504580231	0.132432032	0.700600118	1.047880062
ELN	1.160155767	1.503952409	0.132593621	0.955956412	1.407973615
SRSF7	0.868890478	-1.490259435	0.136156034	0.722258878	1.045290942
VWF	1.15084327	1.464264764	0.143121621	0.953551431	1.388955216
HMGCL	0.869252463	-1.454597853	0.145780626	0.719696734	1.049886445

FSCN1	1.137284978	1.446187859	0.148124518	0.955326311	1.353900867
FECH	0.864932354	-1.444032465	0.148729843	0.710311644	1.053210916
GNAI2	1.149410895	1.434383244	0.151462927	0.950256587	1.390303866
HNRNPM	0.873120953	-1.425472854	0.154020562	0.724526466	1.052190961
VDAC1	0.872094464	-1.422623987	0.154845187	0.722232922	1.053051905
DCTN1	1.161849554	1.419392341	0.155784664	0.944469293	1.429262333
CA4	0.865916945	-1.408578632	0.158959801	0.708724869	1.057973536
NOD2	0.865199421	-1.398334342	0.16201269	0.706278003	1.059880153
ERP29	1.161396981	1.393799737	0.163378088	0.941032694	1.433364597
PNP	0.875410539	-1.393517823	0.163463259	0.725994627	1.055577524
AR	1.105388389	1.383208057	0.16660108	0.959081698	1.274013978
SETD2	0.867214719	-1.382104157	0.166939722	0.708571918	1.061376199
INPP5K	1.14697852	1.378876659	0.167932786	0.943848225	1.393825502
PTMA	1.152513524	1.357162319	0.174729641	0.938899477	1.414728046
CYP2E1	1.056227798	1.351818851	0.176433272	0.97569062	1.143412818
NR1I3	1.081628477	1.349633515	0.177133568	0.965137557	1.212179708
SLC35C1	1.166794111	1.346666641	0.178087621	0.932158077	1.460491018
HNRNPL	0.880734625	-1.346402763	0.17817266	0.732075403	1.0595814
RPSA	0.875209274	-1.34519287	0.178562958	0.720722684	1.062809996
PDHX	0.879766981	-1.342474256	0.179442272	0.729703528	1.060690967
AGER	1.13196403	1.327440125	0.184363134	0.94264639	1.359303531
LGALS1	1.136338499	1.327050029	0.184492134	0.940863316	1.3724259
FH	0.880521315	-1.319992185	0.186837627	0.728933618	1.063632911
NR4A1	1.132102652	1.316662766	0.18795169	0.941178985	1.361756303
SLC26A6	0.87886105	-1.31571839	0.18826858	0.725071434	1.06526986
F5	1.120535334	1.313624063	0.188972747	0.945542902	1.327913763
PRND	1.122683809	1.31291369	0.189212033	0.944563907	1.334392438
DECR1	0.884570882	-1.303704139	0.192334478	0.73561657	1.063686814
GK	0.873526908	-1.302111392	0.192878309	0.712662958	1.070701444
SKIV2L	1.137054129	1.285165174	0.198734571	0.93478368	1.383092282
ATP6V0A1	0.882212483	-1.282729023	0.199587028	0.728468868	1.068403743
PDHA1	0.88234801	-1.279238031	0.200813247	0.728371549	1.068874823
NT5C2	0.881921851	-1.266690065	0.205266134	0.726095056	1.071190536
ALDOB	0.881832737	-1.253574896	0.209996561	0.724431931	1.073432773
MLX	0.884916971	-1.241394953	0.214459883	0.729575273	1.073334137
CYBB	0.88342799	-1.229615297	0.218841199	0.725053161	1.076396954
SOD2	0.880867396	-1.228596933	0.219222966	0.719493809	1.078435087
HNRNPA0	0.889136869	-1.221402418	0.221933692	0.736341738	1.073637867
IGFBP1	1.091682602	1.220808378	0.222158581	0.948274354	1.256778589
SREBF1	1.131232321	1.212058412	0.225490022	0.926735161	1.380854659
KRT19	1.133570823	1.210351484	0.22614405	0.925290416	1.388734594
GMDS	0.89062145	-1.209517282	0.226464176	0.738198818	1.07451617
PGM3	0.890227693	-1.191393295	0.233499224	0.735233418	1.077896253
KLF2	1.115847159	1.183995595	0.236414807	0.930680832	1.337853794
XYLB	0.889294714	-1.178789149	0.238482146	0.731685404	1.08085399
GNAI1	1.123253263	1.178030052	0.238784626	0.925752146	1.362889514
COLQ	1.107900441	1.16274132	0.244934471	0.932155763	1.316779271
LIN7B	1.117343615	1.157927859	0.246893477	0.926026798	1.34818642
AGL	0.888895514	-1.155950347	0.247701467	0.727989108	1.085366837
MAGED2	1.127347191	1.149042596	0.250538417	0.918884627	1.38310257
PPARA	0.891166733	-1.139564917	0.254467608	0.730957687	1.086489903
CYP3A4	1.075135468	1.138307553	0.254992081	0.949050124	1.217971785
RPL35	1.134174928	1.136208017	0.255869516	0.912760255	1.409299716
RPLP1	1.143396363	1.134697622	0.256502034	0.907138131	1.441186517
PMM1	0.894881891	-1.121241719	0.262184986	0.736971611	1.086627473
HP	1.052646327	1.11590494	0.264462827	0.961935076	1.151911722
TJP2	0.830122243	-1.115283127	0.264729114	0.598473326	1.151434674
TRIM21	0.896713055	-1.1147961	0.264937808	0.740307003	1.086163306
PPP2R1B	0.899119179	-1.090906022	0.275314237	0.74275155	1.088406074
MTOR	0.899464291	-1.089927071	0.27574527	0.743422005	1.088259436
MATR3	0.719601214	-1.082260869	0.279136628	0.396539701	1.305861445
RPS14	0.895442961	-1.071174899	0.284090796	0.731610857	1.095962543
TPM3	0.904146899	-1.065166489	0.286800596	0.751132207	1.088332531
RPL28	1.120936761	1.06390217	0.28737302	0.908323948	1.383316188
CYP2D6	1.089873389	1.062433351	0.288039	0.929876289	1.277400036
RPL27A	1.113607291	1.054185776	0.291797871	0.911689269	1.36024547
MPI	1.105823951	1.049569933	0.293915887	0.916446025	1.334335659
ZNF638	1.119157681	1.045407498	0.295834673	0.906208889	1.38214702
FOLR1	1.097633938	1.041697268	0.297552056	0.921162563	1.307912751

NNT	0.90010736	-1.041203462	0.297781129	0.738342409	1.097313726
CFH	1.097083738	1.032846141	0.301675907	0.920194019	1.307977127
ALDOC	1.105891664	1.028474473	0.303726693	0.912871197	1.33972501
TXNDC5	0.895207874	-1.026987088	0.304426544	0.724723857	1.105796547
NR1H3	1.106677646	1.022763624	0.306419621	0.911300527	1.343942394
FGFR1	1.102791353	1.022181051	0.306695218	0.914145201	1.330367174
EGF	0.901710982	-1.017319806	0.309001318	0.738754817	1.100612378
TAT	1.042158123	0.998987456	0.317800768	0.961056116	1.1301042
FKTN	0.899708525	-0.996377471	0.319066775	0.730829422	1.107611989
MSR1	1.102292318	0.982689836	0.325760115	0.907687428	1.338619789
HADHB	0.911752258	-0.978995033	0.327582434	0.757791349	1.096993494
NR1H4	1.082827806	0.970127068	0.331983158	0.922016016	1.271687298
MYO1C	1.105029973	0.969975978	0.332058466	0.903090079	1.352125628
IL1RN	0.910426816	-0.955983901	0.339080368	0.751084807	1.103573098
PDE1A	1.100617806	0.951550679	0.341324905	0.903390159	1.340904086
MLXIPL	1.09303451	0.94893427	0.342654044	0.909574004	1.313498885
RPS27A	1.110627101	0.947375027	0.343447712	0.893912429	1.379880755
TSC1	1.094961868	0.937113183	0.348700341	0.905724974	1.323736815
KPNA6	0.914024284	-0.935098417	0.349737582	0.757051182	1.103545457
TGM2	1.092283865	0.921073308	0.357012155	0.905234017	1.317984099
HMGCS2	0.915284723	-0.905906004	0.364985606	0.755755887	1.108487725
RPL7	1.098829599	0.897433258	0.369487772	0.89441659	1.349959851
KNG1	1.047918371	0.881161018	0.378230675	0.944306883	1.162898346
SLC25A13	0.915877338	-0.878642347	0.379595227	0.752850649	1.114206783
ANKHD1	0.874907311	-0.875459956	0.381323689	0.648675833	1.180039033
EEF1A1	0.916998047	-0.87066018	0.383939727	0.754492694	1.114504388
C3	1.08335198	0.868527491	0.385105629	0.90428796	1.297873647
LPL	0.919082861	-0.861100298	0.389182805	0.758482644	1.113688378
NR1I2	0.924776325	-0.850890619	0.394830117	0.772333273	1.107308569
GRHPR	0.917907152	-0.84986131	0.395402198	0.753363603	1.118388965
PRKCA	0.918272213	-0.845369698	0.397904458	0.753566974	1.118976662
RETN	1.069624721	0.842030714	0.399770761	0.914514301	1.25104336
DPAGT1	0.92021366	-0.825944768	0.408835424	0.755434184	1.120935747
CPS1	0.918049644	-0.820190527	0.412107502	0.748392621	1.126167102
MYH10	1.084299278	0.819344417	0.412589937	0.893446197	1.315921348
PKLR	1.078010459	0.814260278	0.41549586	0.899700357	1.29165954
SLC9A3R1	0.923195778	-0.813931162	0.415684388	0.761588797	1.119095302
TUBA1A	1.080117673	0.809800582	0.418054798	0.896315457	1.301611144
AOX1	1.065377401	0.80783782	0.41918395	0.913639673	1.242315806
RBP4	1.077128885	0.80734919	0.419465331	0.899358718	1.290037682
RPL21	1.085040581	0.798966562	0.424309801	0.888162836	1.325559925
SLC37A4	0.927154057	-0.790813474	0.429052846	0.768671337	1.118312346
ACSS3	0.923200391	-0.790297226	0.429354207	0.757231776	1.125545691
HNRNPK	0.925678632	-0.786661736	0.431479909	0.763653035	1.122081482
RPL8	1.094466399	0.784954923	0.432479998	0.873609558	1.371157959
FHIT	0.931918301	-0.784189062	0.432929182	0.781344043	1.111510004
PPARG	0.927135271	-0.779034656	0.4359593	0.766441178	1.121520915
RPS19	1.090213759	0.777951606	0.436597544	0.877011195	1.355246145
NR2C1	0.926322305	-0.775003237	0.438337747	0.763315975	1.124138682
INVS	0.924241141	-0.759917873	0.447303677	0.754291933	1.132481537
NCL	1.082573785	0.746787092	0.455192088	0.879068666	1.333190507
CTNS	1.073560518	0.741702323	0.458267712	0.889952391	1.29504926
SLC2A10	1.071966052	0.740413506	0.459049126	0.891842562	1.288468688
AGT	0.934229326	-0.727003915	0.467223557	0.777674195	1.12230088
TBP	0.931352562	-0.725623792	0.468069431	0.768579914	1.128597793
IDH1	0.93161205	-0.723829865	0.469170192	0.769007989	1.128598174
EPM2A	0.915333469	-0.712966246	0.475866644	0.717728	1.167343839
ANXA2	1.070868383	0.711744066	0.476623275	0.886850413	1.293069357
ADPRH	1.070566382	0.711483734	0.476784527	0.887228245	1.291789779
KITLG	0.930934673	-0.710534448	0.477372777	0.764160908	1.134105861
EWSR1	0.93317528	-0.705277519	0.480637548	0.770000709	1.130928962
EPAS1	0.93836502	-0.704990729	0.480816006	0.78625229	1.119906322
NR5A2	0.936061257	-0.700813175	0.483419615	0.778127803	1.126049826
TCF7L2	0.930042394	-0.68135338	0.495647915	0.754915837	1.145795085
TF	1.034403274	0.67420935	0.500178226	0.937530859	1.141285243
KAT5	0.934541872	-0.673568314	0.500585804	0.767443478	1.138023237
LIPG	0.937965801	-0.671319968	0.502016716	0.778009829	1.130808136
ETFDH	0.939383611	-0.662754716	0.50748766	0.780785145	1.130197691
POMT2	1.066569279	0.654303526	0.512916274	0.879321932	1.293690042

SLC6A4	0.937415762	-0.654111524	0.513039957	0.77237948	1.137715764
EPCAM	0.937242536	-0.648815322	0.516457753	0.770586686	1.139941279
ASS1	1.064871838	0.645377478	0.518682595	0.879827072	1.288835122
TUFM	0.934390583	-0.643686406	0.519778807	0.759959399	1.14885843
SORD	0.939685195	-0.638077515	0.523423222	0.776234904	1.137552899
P4HB	0.937363033	-0.635611906	0.525029407	0.767861998	1.144280427
PLCB1	1.062477246	0.617402323	0.53696939	0.876530599	1.287870497
RPL34	1.061696944	0.607194281	0.543721987	0.875131405	1.288035595
SLC26A1	1.059100195	0.597300313	0.550306896	0.877221645	1.278688494
PRDX6	1.061576681	0.588253001	0.556362487	0.869932581	1.295439526
HNRNPA1	0.943993742	-0.584804218	0.558679368	0.778177051	1.145143233
PNPLA3	0.943117175	-0.581367599	0.56099273	0.774138862	1.148979918
NAV2	0.944432027	-0.581182206	0.561117659	0.778819884	1.14526076
GAPDH	0.942025524	-0.575908647	0.564676931	0.768760964	1.154340725
ATF3	1.05382328	0.5657874	0.571538313	0.878814258	1.263683986
KHK	0.945936695	-0.555632201	0.578462314	0.777529327	1.150819912
DPYSL2	0.950183474	-0.553563003	0.579877952	0.792924535	1.138631224
EP300	0.947282375	-0.5318925	0.594800452	0.775905631	1.156511645
RPL24	1.059398807	0.53041	0.595827694	0.855972917	1.311169793
GBE1	0.950225407	-0.523971922	0.600298069	0.785028075	1.150186027
EHHADH	0.953414036	-0.514433079	0.606949237	0.794959975	1.143451688
HSD11B1	1.048638781	0.510354712	0.609802979	0.87380443	1.258454701
AHR	1.053028403	0.507653127	0.611696626	0.862586509	1.285516067
TPM4	0.949765572	-0.506298041	0.612647438	0.77797541	1.159489915
FAH	1.049050598	0.495428741	0.620297511	0.868007996	1.267853709
SPTAN1	0.953458291	-0.49499188	0.620605852	0.789486947	1.151485424
RPS15A	0.950836451	-0.487297333	0.626047638	0.776327568	1.164572783
RBKS	0.953434177	-0.486626039	0.626523367	0.786829748	1.155315661
WDR26	1.050724848	0.482537256	0.629424339	0.859419554	1.284614368
RPS3A	0.951572295	-0.476308631	0.633854517	0.775767951	1.167217375
MGST2	0.953073608	-0.474996235	0.634789657	0.78162143	1.162134593
EDN1	0.955222103	-0.472544677	0.63653806	0.78992095	1.155114655
ABCC2	0.956735651	-0.472090962	0.636861864	0.796244747	1.149575063
KLF9	1.047185917	0.465937344	0.64126035	0.862568848	1.27131689
KRT7	1.041108512	0.460534866	0.645132351	0.877073084	1.235822823
CKB	0.957119206	-0.458251809	0.646771542	0.793518168	1.154450159
TPPP3	1.041778214	0.448439614	0.653835958	0.871136327	1.245846159
OXA1L	1.047782691	0.444834926	0.656439053	0.853012224	1.287025598
AZGP1	1.044833997	0.443966494	0.657066809	0.860914137	1.268045249
HLCS	0.957595677	-0.443415425	0.657465281	0.790686857	1.159737855
ABAT	0.960064686	-0.429414078	0.66762191	0.79710418	1.156340945
HBB	0.958396185	-0.425369437	0.670567382	0.787972012	1.165680042
GLS2	1.044670325	0.423287709	0.672085361	0.853294469	1.278967727
GPT	1.043570249	0.418256868	0.675759322	0.85453235	1.274426725
TRAF6	0.958592229	-0.41632064	0.677175391	0.78554229	1.169763962
HGF	0.954307128	-0.412140552	0.680236407	0.764000759	1.192017263
DERA	0.958931222	-0.407882688	0.683359803	0.783920976	1.17301248
F2	1.028913284	0.407633621	0.683542677	0.897139045	1.180042885
H6PD	1.040565096	0.403895765	0.686289371	0.857961188	1.262033451
HNF1A	1.041875952	0.396665536	0.691614118	0.850717393	1.275988369
FAM120A	0.960581841	-0.393356491	0.6940562	0.786156453	1.173707178
PCK1	0.962645631	-0.389613703	0.696822218	0.794865909	1.165840175
KPNA1	0.960716166	-0.387248034	0.698572593	0.784339925	1.17675452
NEAT1	0.957503034	-0.382749959	0.701905168	0.766591091	1.195959711
AMBP	0.96640183	-0.379901706	0.704018381	0.810185651	1.152738876
GTF2I	0.965148178	-0.369611208	0.711672199	0.79964912	1.164899682
YWHAB	0.96583446	-0.367860337	0.712977376	0.802535207	1.16236172
ST6GAL1	0.96678566	-0.365996003	0.714368058	0.806809868	1.158481756
CIRBP	1.036066998	0.36214861	0.71724098	0.855279732	1.255068704
GLS	1.036350469	0.359145082	0.719486558	0.85286865	1.259305632
CHKA	0.965864052	-0.359028182	0.719574007	0.799044995	1.16751043
IQGAP1	1.036661834	0.357661405	0.720596719	0.85103348	1.262779646
ITPR2	0.963356902	-0.352537641	0.724435094	0.78279997	1.18556024
MST1	1.031434716	0.351602147	0.725136655	0.867985264	1.225663173
MTMR1	0.969462549	-0.329948782	0.741438663	0.806348181	1.165572958
EIF4G3	0.968266114	-0.324941998	0.74522497	0.797110771	1.176171872
LIN7A	0.971675991	-0.323610669	0.74623281	0.816478309	1.156373931
KYNU	1.030733839	0.317789247	0.750644808	0.855195358	1.24230357
HNF4A	0.97054656	-0.315853054	0.752114047	0.806211597	1.168378907

RPS4X	1.031571609	0.30382989	0.761257493	0.844144449	1.260613614
MYL6	1.030948183	0.302217478	0.762486282	0.846042699	1.256265385
CYP4F2	0.970513434	-0.298422938	0.765380384	0.797317199	1.181332005
ARG1	1.019965484	0.29724043	0.766282957	0.895311143	1.161975472
ABCC3	0.970323233	-0.29580073	0.767382265	0.794740252	1.184697985
APOA1	1.022521137	0.288803596	0.77273168	0.879085918	1.189359827
PC	0.972359815	-0.288186404	0.773204056	0.803596608	1.176564957
THRB	1.026150971	0.276058108	0.782503436	0.854305661	1.232563313
KDELR2	1.026753929	0.275940698	0.782593614	0.851182136	1.238540597
LMO7	0.975947249	-0.253492068	0.79988799	0.808485745	1.178095023
LGALS3	0.97639061	-0.249830061	0.802718772	0.809503501	1.177683137
RNF31	1.02508412	0.242058616	0.808734744	0.838762688	1.252794704
RANBP10	0.976413276	-0.238145172	0.811768501	0.802264461	1.188364849
RPL22	1.022390098	0.22737741	0.820130285	0.844738876	1.237401927
HKDC1	0.980402489	-0.220611751	0.825394751	0.822316917	1.168879079
MKLN1	0.976815555	-0.208496791	0.834841088	0.783512797	1.217808608
AKR1B1	1.019123842	0.202095329	0.8398422	0.848086436	1.22465513
MYH9	0.979485458	-0.197724333	0.84326075	0.797562298	1.20290511
LDHA	0.981493466	-0.191956448	0.84777632	0.811062177	1.187738068
DOLPP1	1.018133171	0.189664108	0.849572348	0.845576706	1.225903157
RPS3	0.980879013	-0.183279342	0.854578843	0.797906752	1.20580962
MYH14	0.982559966	-0.182897585	0.854878378	0.813724533	1.186426178
RPS9	1.020612581	0.180548516	0.85672197	0.817843615	1.273654303
ACSL1	0.982810982	-0.178889671	0.858024333	0.812773013	1.188422119
RPL3	1.01905793	0.175883593	0.860385396	0.825723207	1.257660021
ERN1	1.017174332	0.166687356	0.867616055	0.832602994	1.242661424
RPS16	0.9837574	-0.156608406	0.875553478	0.8014585	1.207521814
GID4	1.014944818	0.152203888	0.879026127	0.838458924	1.228578949
PGAM1	0.98671665	-0.143307512	0.886047323	0.821797519	1.184731913
ALDH18A1	0.986138053	-0.142018302	0.88706555	0.813342502	1.195644218
PHKA2	0.986252161	-0.136692508	0.891273852	0.808694541	1.202794474
IKBKB	1.013239284	0.136313873	0.891573157	0.83865395	1.224168618
PROS1	0.988467872	-0.123041819	0.902073983	0.821712481	1.189064005
PDE5A	0.987143105	-0.12210769	0.902813731	0.802000703	1.215025756
ANPEP	0.989016979	-0.114569277	0.908786533	0.818753392	1.194687674
PIK3CG	0.988026418	-0.113518347	0.909619619	0.802498959	1.216445443
MDH2	0.988741588	-0.101815503	0.91890312	0.795107742	1.229531391
DCN	0.99047546	-0.100802309	0.919707394	0.822300399	1.193045314
NLRP3	0.989433576	-0.100443347	0.919992358	0.804202898	1.21732812
STUB1	0.989583982	-0.098924919	0.921197883	0.804188353	1.217720269
MYO1E	1.009047677	0.091403032	0.927172352	0.831826985	1.224025229
KLKB1	0.9924894	-0.091206761	0.927328303	0.844048621	1.167036098
VCL	1.008914423	0.087255119	0.930468728	0.826564247	1.231493276
ME1	0.99200969	-0.084583246	0.932592719	0.823725755	1.194673373
PRKAA1	0.991556881	-0.083547379	0.933416306	0.812702947	1.209771728
ABCA1	1.008548612	0.081232919	0.935256721	0.821297807	1.238491438
PLA2G1B	1.008271005	0.073891489	0.941096728	0.810383039	1.254481364
LDLR	0.995178005	-0.050154279	0.959999446	0.823883013	1.202087246
MACF1	0.995190125	-0.04522618	0.963927027	0.807534402	1.22645349
DLGAP4	1.004393334	0.044962716	0.964137027	0.829687548	1.215886597
LPIN1	0.995819564	-0.042940765	0.965748753	0.822506743	1.205651641
RPS26	1.00379618	0.037169422	0.970349919	0.822006762	1.225788908
NCOA2	1.003059368	0.030859721	0.975381412	0.826169442	1.217822936
HMGB2	0.997291027	-0.028074333	0.977602865	0.825231075	1.205225327
BBS4	0.998017321	-0.020186576	0.983894537	0.823098901	1.21010801
MTTP	0.998373397	-0.017082855	0.986370517	0.828279525	1.203397417
RPS10	0.998570729	-0.014227952	0.98864812	0.819992949	1.216039117
APOB	0.999813653	-0.002503615	0.998002406	0.864086821	1.15685984
RPS12	1.000146495	0.001437094	0.998853365	0.819030371	1.221313697
