# Supplementary material for: Effect of aberrant fructose metabolism following SARS-CoV-2 infection on colorectal cancer patients’ poor prognosis
Source: PLoS Comput Biol. 2024 Sep 27;20(9):e1012412. doi: 10.1371/journal.pcbi.1012412 (PMC11463760; doi:10.1371/journal.pcbi.1012412)
Supplement: S2 Table — (PDF) [file pcbi.1012412.s002.pdf]

Supplementary Table S2

	logo	geneSet	motif	NES	AUC	TF_highConf	TF_lowConf	nEnrGenes	rankAtMax	enrichedGenes
1	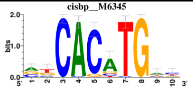	Module_Gene	cisbp__M6345	5.22	0.209	MITF (directAnnotation).	ARNTL; TFEC (inferredBy_MotifSimilarity).	4	429	ALDH3B1;FTCD;PFKFB1;PTPRD
2	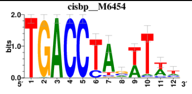	Module_Gene	cisbp__M6454	4.54	0.186	RORA (directAnnotation).	NR1D1; RORB; RORC (inferredBy_MotifSimilarity).	4	654	AOC3;GATM;IL7;PTPRD
3	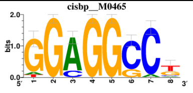	Module_Gene	cisbp__M0465	4.25	0.176			4	855	ALDH3B1;FTCD;PFKFB1;TIMP1
4	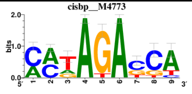	Module_Gene	cisbp__M4773	4.14	0.172			5	1185	AOC3;GATM;IL7;PFKFB1;SERPINE1
5	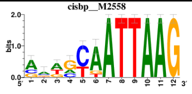	Module_Gene	cisbp__M2558	4.06	0.169			3	429	GPI;IL7;SERPINE1
6	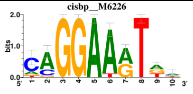	Module_Gene	cisbp__M6226	3.95	0.166	FEV (directAnnotation).	ELK3 (inferredBy_MotifSimilarity).	4	654	IL7;PFKFB1;PTPRD;SERPINE1
7	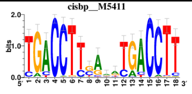	Module_Gene	cisbp__M5411	3.94	0.165	ESRRG (directAnnotation).	ESRRA; RARA; RARB; RARG; RXRA (inferredBy_MotifSimilarity). NR5A2 (inferredBy_MotifSimilarity_n_Orthology).	3	429	FTCD;GPI;PFKFB1
8	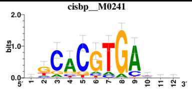	Module_Gene	cisbp__M0241	3.89	0.163		BHLHE40 (inferredBy_MotifSimilarity).	4	855	ALDH3B1;IL7;PFKFB1;PTPRD
9	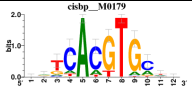	Module_Gene	cisbp__M0179	3.87	0.163	ARNT (inferredBy_Orthology).	TFE3 (inferredBy_MotifSimilarity).	5	1221	ALDH3B1;FTCD;GPI;PFKFB1;PTPRD
10	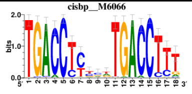	Module_Gene	cisbp__M6066	3.87	0.163	RARA (inferredBy_Orthology).	ESRRA; NR1H3; NR1I2; NR2C1; NR2F2; PPARA; RARG (inferredBy_MotifSimilarity). NR2F6; RARB (inferredBy_MotifSimilarity_n_Orthology).	3	321	FTCD;PFKFB1;SERPINE1
11	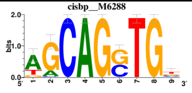	Module_Gene	cisbp__M6288	3.7	0.157	TCF12 (directAnnotation).	ASCL2; MYF6 (inferredBy_MotifSimilarity). ASCL1 (inferredBy_MotifSimilarity_n_Orthology).	3	290	IL7;PFKFB1;PTPRD

12	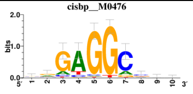	Module_Gene	cisbp__M0476	3.67	0.156			3	429	ALDH3B1;FTCD;PFKFB1
13	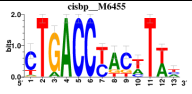	Module_Gene	cisbp__M6455	3.67	0.156	RORC (directAnnotation).	NR1D1; RORA; RORB (inferredBy_MotifSimilarity).	2	69	IL7;PTPRD
14	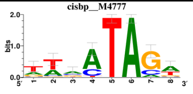	Module_Gene	cisbp__M4777	3.51	0.151			8	3325	AASS;ALDH3B1;CLEC4A;CTNNB1;IL7;PTPRD;SERPINE1;TIMP1
15	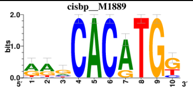	Module_Gene	cisbp__M1889	3.49	0.15	MAX (directAnnotation).	MYC (inferredBy_MotifSimilarity).	4	687	ALDH3B1;FTCD;GATM;PFKFB1
16	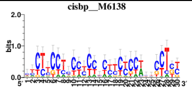	Module_Gene	cisbp__M6138	3.47	0.149	RELA (inferredBy_Orthology).	CUX1; KLF15; MAZ; PATZ1; REST; VEZF1; WT1; ZNF263; ZNF341; ZNF467 (inferredBy_MotifSimilarity). BCL6; ELF5; SP5 (inferredBy_MotifSimilarity_n_Orthology).	4	855	AOC3;IL7;PFKFB1;PTPRD
17	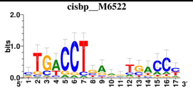	Module_Gene	cisbp__M6522	3.41	0.147	THRA (directAnnotation).	NR1H3; RARA; THRB (inferredBy_MotifSimilarity). RXRA (inferredBy_MotifSimilarity_n_Orthology).	3	429	AOC3;FTCD;PTPRD
18	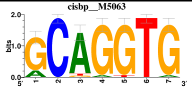	Module_Gene	cisbp__M5063	3.41	0.147	TCF12; TCF3; TCF4 (inferredBy_Orthology).	SNAI1 (inferredBy_MotifSimilarity). ASCL1; ASCL2 (inferredBy_MotifSimilarity_n_Orthology).	3	496	IL7;PFKFB1;PTPRD
19	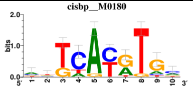	Module_Gene	cisbp__M0180	3.37	0.146	ARNT2 (inferredBy_Orthology).		3	463	ALDH3B1;FTCD;PFKFB1
20	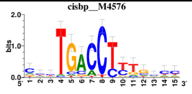	Module_Gene	cisbp__M4576	3.31	0.144	RXRA (directAnnotation).	ESRRA; HNF4A; HNF4G; NR1H2; NR1H3; NR1I2; NR1I3; NR2C2; NR2F1; NR2F2; NR2F6; NR4A3; PPARA; PPARD; PPARG; RARA; RARB; RARG; RXRB; RXRG; ZNF71 (inferredBy_MotifSimilarity). NR2C1 (inferredBy_MotifSimilarity_n_Orthology).	3	654	AOC3;PFKFB1;SERPINE1
21	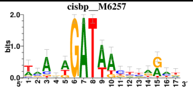	Module_Gene	cisbp__M6257	3.31	0.144	GATA5 (directAnnotation).		4	855	AOC3;IL7;PFKFB1;SERPINE1
22	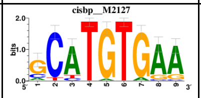	Module_Gene	cisbp__M2127	3.27	0.142			3	654	ALDH3B1;AOC3;GATM
23	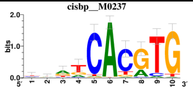	Module_Gene	cisbp__M0237	3.25	0.142		TFE3 (inferredBy_MotifSimilarity).	4	855	ALDH3B1;FTCD;PFKFB1;PTPRD
24	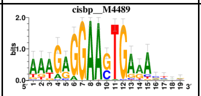	Module_Gene	cisbp__M4489	3.24	0.141	SPI1 (directAnnotation).	BCL11A; EP300; IKZF1; IRF1; IRF2; IRF3; IRF4; IRF5; IRF6; IRF7; IRF8; MTA3; PAX5; PRDM1; SPIB; STAT1; STAT2; STAT3; TBL1XR1 (inferredBy_MotifSimilarity).	3	697	IL7;PTPRD;TIMP1

25	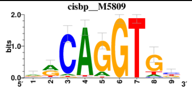	Module_Gene	cisbp__M5809	3.22	0.141	SNAI2 (directAnnotation).	ASCL1; ID4; MYF5; MYF6; MYOD1; MYOG; SNAI1; SNAI3; TCF12; TCF3; TCF4; TFF3 (inferredBy_MotifSimilarity). ASCL2; MSC; TCF21; TCF23; TCF24 (inferredBy_MotifSimilarity_n_Orthology).	3	654	IL7;PFKFB1;PTPRD
26	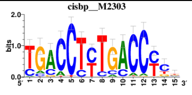	Module_Gene	cisbp__M2303	3.22	0.141	NR2C2 (directAnnotation).	HDAC2; HNF4A; HNF4G; NR1H2; NR1H4; NR2E3; NR2F1; NR2F2; NR2F6; PPARA; PPARD; PPARG; RXRA; RXRB; RXRG; SP1; TCF12; THRB (inferredBy_MotifSimilarity). NR1H3 (inferredBy_MotifSimilarity_n_Orthology).	9	4340	AASS;AOC3;CLEC4A;FTCD;GPI;IL7;PFKFB1;SERPINE1;TIMP1
27	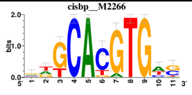	Module_Gene	cisbp__M2266	3.2	0.14	BHLHE40 (inferredBy_Orthology).		7	2213	ALDH3B1;FTCD;GPI;IL7;PFKFB1;PTPRD;SERPINE1
28	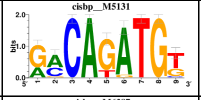	Module_Gene	cisbp__M5131	3.16	0.139	TCF12; TCF3; TCF4 (inferredBy_Orthology).	ATOH7; BHLHE22; BHLHE23 (inferredBy_MotifSimilarity_n_Orthology).	8	4998	AASS;ALDH3B1;AOC3;FTCD;GATM;PFKFB1;PTPRD;SERPINE1
29	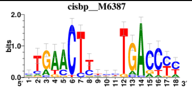	Module_Gene	cisbp__M6387	3.15	0.138	NR1I3 (directAnnotation).	NR1H2; NR1H3; NR1I2; NR2F1; NR2F2; RARA; RARB; RARG; RXRA; RXRB (inferredBy_MotifSimilarity).	4	1054	AOC3;FTCD;PFKFB1;SERPINE1
30	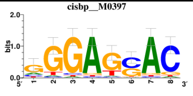	Module_Gene	cisbp__M0397	3.13	0.138	ZNF263 (inferredBy_Orthology).		2	168	FTCD;GATM
31	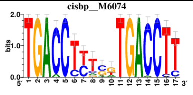	Module_Gene	cisbp__M6074	3.13	0.138	RARG (inferredBy_Orthology).	NR1I2; NR1I3; RARA; RXRA; RXRB; THRB (inferredBy_MotifSimilarity). RARB (inferredBy_MotifSimilarity_n_Orthology).	3	687	FTCD;PFKFB1;SERPINE1
32	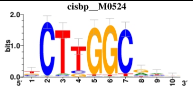	Module_Gene	cisbp__M0524	3.09	0.137			4	923	ALDH3B1;AOC3;IL7;PTPRD
33	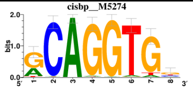	Module_Gene	cisbp__M5274	3.06	0.136	SNAI1; SNAI2; SNAI3 (inferredBy_Orthology).	MYOD1 (inferredBy_MotifSimilarity).	3	600	IL7;PFKFB1;PTPRD
34	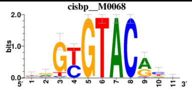	Module_Gene	cisbp__M0068	3.06	0.136			2	168	PFKFB1;SERPINE1
35	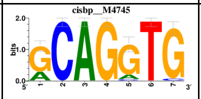	Module_Gene	cisbp__M4745	3.05	0.135	ASCL1; ASCL2 (inferredBy_Orthology).	SNAI1; TCF12; TCF4 (inferredBy_MotifSimilarity). SNAI2; SNAI3; TCF3 (inferredBy_MotifSimilarity_n_Orthology).	2	191	IL7;PTPRD
36	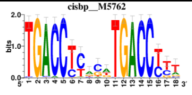	Module_Gene	cisbp__M5762	3.04	0.135	RARA (directAnnotation).	ESRRA; NR1I2; NR2C1; NR2C2; RARG; RXRA; RXRB; THRB (inferredBy_MotifSimilarity). NR2F6; NR5A1; RARB; THRA (inferredBy_MotifSimilarity_n_Orthology).	5	1588	AOC3;FTCD;GPI;PFKFB1;SERPINE1
37	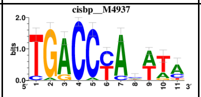	Module_Gene	cisbp__M4937	3.01	0.134	NR1D1; NR1D2 (inferredBy_Orthology).	RORA; RORC (inferredBy_MotifSimilarity).	6	2002	ALDH3B1;AOC3;GATM;IL7;PTPRD;SERPINE1
