# Supplementary material for: Effect of aberrant fructose metabolism following SARS-CoV-2 infection on colorectal cancer patients’ poor prognosis
Source: PLoS Comput Biol. 2024 Sep 27;20(9):e1012412. doi: 10.1371/journal.pcbi.1012412 (PMC11463760; doi:10.1371/journal.pcbi.1012412)
Supplement: S3 Table — 15 mRNA-miRNA relationship pairs linked with mRNAs were retrieved from the miRWalk database. (PDF) [file pcbi.1012412.s003.pdf]

Table S2	
mRNA	miRNA
GPI	hsa-miR-551a
GPI	hsa-miR-600
GPI	hsa-miR-612
GPI	hsa-miR-320b
GPI	hsa-miR-297
GPI	hsa-miR-646
GPI	hsa-miR-760
GPI	hsa-miR-1303
GPI	hsa-miR-1269a
GPI	hsa-miR-1273c
GPI	hsa-miR-1260b
GPI	hsa-miR-4478
PMM2	hsa-miR-198
PMM2	hsa-miR-326
PMM2	hsa-miR-422a
PMM2	hsa-miR-492
PMM2	hsa-miR-551a
PMM2	hsa-miR-646
PMM2	hsa-miR-320b
PMM2	hsa-miR-1303
PMM2	hsa-miR-2110
PMM2	hsa-miR-1260b
PMM2	hsa-miR-4286
SERPINE1	hsa-miR-107
SERPINE1	hsa-miR-326
SERPINE1	hsa-miR-484
SERPINE1	hsa-miR-551a
SERPINE1	hsa-miR-612
SERPINE1	hsa-miR-1275
SERPINE1	hsa-miR-4286
ALDH3B1	hsa-miR-1273c
ALDH3B1	hsa-miR-4301
ALDH3B1	hsa-miR-4286
ALDH3B1	hsa-miR-198
ALDH3B1	hsa-miR-622
ALDH3B1	hsa-miR-320b
ALDH3B1	hsa-miR-7702
ALDH3B1	hsa-miR-551a
ALDH3B1	hsa-miR-1303
PTPRD	hsa-miR-320b
PTPRD	hsa-miR-1260b
PTPRD	hsa-miR-567
PTPRD	hsa-miR-650
PTPRD	hsa-miR-1246
PTPRD	hsa-miR-1275
CTNNB1	hsa-miR-484
CTNNB1	hsa-miR-1275
CTNNB1	hsa-miR-1260b
CTNNB1	hsa-miR-3666
CTNNB1	hsa-miR-4286
PFKFB1	hsa-miR-612
PFKFB1	hsa-miR-198
PFKFB1	hsa-miR-422a
PFKFB1	hsa-miR-1258
AOC3	hsa-miR-198
AOC3	hsa-miR-422a
AOC3	hsa-miR-492
AOC3	hsa-miR-650
AOC3	hsa-miR-320b
AOC3	hsa-miR-760
AOC3	hsa-miR-551a
AOC3	hsa-miR-646
FDFT1	hsa-miR-1269a
FDFT1	hsa-miR-320e
FDFT1	hsa-miR-320b
FTCD	hsa-miR-622
FTCD	hsa-miR-760

FTCD	hsa-miR-492
FTCD	hsa-miR-1246
GATM	hsa-miR-2110
TIMP1	hsa-miR-198
TIMP1	hsa-miR-612
AASS	hsa-miR-198
AASS	hsa-miR-326
AASS	hsa-miR-1303
AASS	hsa-miR-3666
AASS	hsa-miR-451a
AASS	hsa-miR-544a
AASS	hsa-miR-608
AASS	hsa-miR-650
AASS	hsa-miR-1273c
CLEC4A	hsa-miR-297
