# Supplementary material for: Effect of aberrant fructose metabolism following SARS-CoV-2 infection on colorectal cancer patients’ poor prognosis
Source: PLoS Comput Biol. 2024 Sep 27;20(9):e1012412. doi: 10.1371/journal.pcbi.1012412 (PMC11463760; doi:10.1371/journal.pcbi.1012412)
Supplement: S4 Table — Interacting lncRNAs were predicted based on miRNAs of S3 Table. A total of 2,926 pairs of interactions were obtained. (PDF) [file pcbi.1012412.s004.pdf]

Table S3		
miRNA	lncRNA	
hsa-miR-107	AL645608.1	
hsa-miR-107	AL390719.2	
hsa-miR-107	LINC01134	
hsa-miR-107	AL603962.1	
hsa-miR-107	LINC01772	
hsa-miR-107	AL137127.1	
hsa-miR-107	LINC01355	
hsa-miR-107	ELOA-AS1	
hsa-miR-107	AL354919.1	
hsa-miR-107	AL031985.3	
hsa-miR-107	AL139220.2	
hsa-miR-107	AL691459.1	
hsa-miR-107	AL139147.1	
hsa-miR-107	LAMTOR5-AS1	
hsa-miR-107	SLC16A1-AS1	
hsa-miR-107	RP6-42F4.1	
hsa-miR-107	LIX1L-AS1	
hsa-miR-107	RP4-565E6.1	
hsa-miR-107	XXyac-YX155B6.6	
hsa-miR-107	AL645568.1	
hsa-miR-107	LINC01351	
hsa-miR-107	MIR181A1HG	
hsa-miR-107	AC119427.1	
hsa-miR-107	MIR29B2CHG	
hsa-miR-107	ACBD3-AS1	
hsa-miR-107	AL162595.1	
hsa-miR-107	AL591848.4	
hsa-miR-107	AC010969.2	
hsa-miR-107	AC013400.1	
hsa-miR-107	AL133243.3	
hsa-miR-107	AC016722.3	
hsa-miR-107	AC007744.1	
hsa-miR-107	AC016747.1	
hsa-miR-107	LINC01798	
hsa-miR-107	PCBP1-AS1	
hsa-miR-107	ALMS1-IT1	
hsa-miR-107	DGUOK-AS1	
hsa-miR-107	TCF7L1-IT1	
hsa-miR-107	CYTOR	
hsa-miR-107	AC062029.1	
hsa-miR-107	AC092168.2	
hsa-miR-107	LINC01885	
hsa-miR-107	LINC01106	
hsa-miR-107	PAX8-AS1	
hsa-miR-107	LINC01101	
hsa-miR-107	AC019080.1	
hsa-miR-107	TTN-AS1	
hsa-miR-107	AC069277.1	
hsa-miR-107	AC018809.2	
hsa-miR-107	AC093495.1	
hsa-miR-107	FGD5-AS1	
hsa-miR-107	LINC00691	
hsa-miR-107	ACVR2B-AS1	
hsa-miR-107	AC012467.2	
hsa-miR-107	AC097634.1	
hsa-miR-107	LINC00960	
hsa-miR-107	DUBR	
hsa-miR-107	AC128688.2	
hsa-miR-107	AC092468.1	
hsa-miR-107	AC083798.2	
hsa-miR-107	LINC02035	
hsa-miR-107	AC097103.2	
hsa-miR-107	AC022498.2	
hsa-miR-107	DLG1-AS1	
hsa-miR-107	CTBP1-AS2	
hsa-miR-107	AFAP1-AS1	
hsa-miR-107	AC107398.3	
hsa-miR-107	AC107068.1	
hsa-miR-107	AC097460.1	
hsa-miR-107	AC108062.1	
hsa-miR-107	LINC02432	

hsa-miR-107	AC093908.1	
hsa-miR-107	MIR4453HG	
hsa-miR-107	AC104083.1	
hsa-miR-107	AC097534.2	
hsa-miR-107	LINC02362	
hsa-miR-107	LINC02434	
hsa-miR-107	SLC9A3-AS1	
hsa-miR-107	AC106744.1	
hsa-miR-107	AC008875.1	
hsa-miR-107	MRPS30-DT	
hsa-miR-107	AC093297.2	
hsa-miR-107	PART1	
hsa-miR-107	CKMT2-AS1	
hsa-miR-107	RGMB-AS1	
hsa-miR-107	LINC01023	
hsa-miR-107	STARD4-AS1	
hsa-miR-107	AC116353.4	
hsa-miR-107	AC005609.1	
hsa-miR-107	AC021078.1	
hsa-miR-107	SAP30L-AS1	
hsa-miR-107	AL008729.1	
hsa-miR-107	CASC15	
hsa-miR-107	AL451165.2	
hsa-miR-107	AL021368.2	
hsa-miR-107	AL135905.1	
hsa-miR-107	AL589935.1	
hsa-miR-107	Z94721.2	
hsa-miR-107	HOXA-AS2	
hsa-miR-107	AC005154.1	
hsa-miR-107	TRG-AS1	
hsa-miR-107	AC004837.2	
hsa-miR-107	LINC01446	
hsa-miR-107	EGFR-AS1	
hsa-miR-107	AC006480.2	
hsa-miR-107	AC114737.1	
hsa-miR-107	AC003092.1	
hsa-miR-107	DLX6-AS1	
hsa-miR-107	STAG3L5P-PVRIG2P-PILRB	
hsa-miR-107	AC069281.2	
hsa-miR-107	SLC26A4-AS1	
hsa-miR-107	AC090114.2	
hsa-miR-107	EPHA1-AS1	
hsa-miR-107	LINC00689	
hsa-miR-107	LINC00599	
hsa-miR-107	AF106564.1	
hsa-miR-107	AC105150.1	
hsa-miR-107	LINC01419	
hsa-miR-107	AF117829.1	
hsa-miR-107	AC018781.1	
hsa-miR-107	AZIN1-AS1	
hsa-miR-107	AC090198.1	
hsa-miR-107	AC108002.1	
hsa-miR-107	AL162231.4	
hsa-miR-107	SMC5-AS1	
hsa-miR-107	AL590705.1	
hsa-miR-107	AL590705.5	
hsa-miR-107	AL512590.3	
hsa-miR-107	AL441992.1	
hsa-miR-107	AL358781.1	
hsa-miR-107	AL359878.1	
hsa-miR-107	LINC00702	
hsa-miR-107	AL137145.1	
hsa-miR-107	AC010997.6	
hsa-miR-107	NUTM2B-AS1	
hsa-miR-107	TMEM254-AS1	
hsa-miR-107	LINC00858	
hsa-miR-107	AC067750.1	
hsa-miR-107	NUTM2A-AS1	
hsa-miR-107	AL391684.1	
hsa-miR-107	RPARP-AS1	
hsa-miR-107	CASC2	
hsa-miR-107	AL391005.1	
hsa-miR-107	AP006621.3	

hsa-miR-107	H19	
hsa-miR-107	KCNQ1OT1	
hsa-miR-107	LINC00294	
hsa-miR-107	AC080100.1	
hsa-miR-107	AP003559.1	
hsa-miR-107	NEAT1	
hsa-miR-107	AP002748.3	
hsa-miR-107	AP000560.1	
hsa-miR-107	AP003119.3	
hsa-miR-107	AP000974.1	
hsa-miR-107	AP000766.1	
hsa-miR-107	COLCA1	
hsa-miR-107	AP001781.1	
hsa-miR-107	AP003393.1	
hsa-miR-107	AP000866.2	
hsa-miR-107	AC092471.2	
hsa-miR-107	AC005831.1	
hsa-miR-107	AC010168.2	
hsa-miR-107	AC084816.1	
hsa-miR-107	DDX11-AS1	
hsa-miR-107	AC015540.1	
hsa-miR-107	AC008124.1	
hsa-miR-107	AC004466.1	
hsa-miR-107	AC117498.1	
hsa-miR-107	AC011603.2	
hsa-miR-107	AC008147.2	
hsa-miR-107	AC090502.1	
hsa-miR-107	AC078923.1	
hsa-miR-107	POC1B-AS1	
hsa-miR-107	RMST	
hsa-miR-107	HELLPAR	
hsa-miR-107	AC079316.2	
hsa-miR-107	AC027290.2	
hsa-miR-107	AC026362.1	
hsa-miR-107	AC055713.1	
hsa-miR-107	AC068790.9	
hsa-miR-107	AC131212.3	
hsa-miR-107	N4BP2L2-IT2	
hsa-miR-107	DLEU2	
hsa-miR-107	AL162377.1	
hsa-miR-107	LINC00355	
hsa-miR-107	AL137782.1	
hsa-miR-107	MIR4500HG	
hsa-miR-107	AL161431.1	
hsa-miR-107	LINC00641	
hsa-miR-107	AL132780.2	
hsa-miR-107	AL135999.2	
hsa-miR-107	LINC02313	
hsa-miR-107	PSMA3-AS1	
hsa-miR-107	AL049775.1	
hsa-miR-107	AL928654.4	
hsa-miR-107	SNHG14	
hsa-miR-107	AC020658.2	
hsa-miR-107	AC090984.1	
hsa-miR-107	MIR9-3HG	
hsa-miR-107	AC091167.5	
hsa-miR-107	AC016251.1	
hsa-miR-107	AC118658.1	
hsa-miR-107	AC023024.2	
hsa-miR-107	Z98883.1	
hsa-miR-107	AC093525.10	
hsa-miR-107	AC141586.4	
hsa-miR-107	ERVK13-1	
hsa-miR-107	MMP25-AS1	
hsa-miR-107	AC012676.5	
hsa-miR-107	AC007221.1	
hsa-miR-107	AC007014.1	
hsa-miR-107	RRN3P2	
hsa-miR-107	AC025279.1	
hsa-miR-107	AC009093.10	
hsa-miR-107	SLX1B-SULT1A4	
hsa-miR-107	AC120114.4	
hsa-miR-107	AC120114.2	

hsa-miR-107	SLX1A-SULT1A3	
hsa-miR-107	AC079416.3	
hsa-miR-107	AC010533.1	
hsa-miR-107	AC040162.3	
hsa-miR-107	AC020978.7	
hsa-miR-107	AC020978.5	
hsa-miR-107	AC009060.1	
hsa-miR-107	AC079414.3	
hsa-miR-107	AC136603.1	
hsa-miR-107	AC027279.1	
hsa-miR-107	AC092718.7	
hsa-miR-107	AC092135.3	
hsa-miR-107	AC092127.1	
hsa-miR-107	AC010536.1	
hsa-miR-107	AC005696.1	
hsa-miR-107	AC104581.4	
hsa-miR-107	AC093484.1	
hsa-miR-107	SMCR5	
hsa-miR-107	AC026271.3	
hsa-miR-107	CCDC144NL-AS1	
hsa-miR-107	AC003102.1	
hsa-miR-107	AC008105.1	
hsa-miR-107	SP2-AS1	
hsa-miR-107	AC018521.1	
hsa-miR-107	AC018628.1	
hsa-miR-107	AC005332.6	
hsa-miR-107	LINC01482	
hsa-miR-107	KCNJ2-AS1	
hsa-miR-107	SOX9-AS1	
hsa-miR-107	AC097641.2	
hsa-miR-107	LINC01979	
hsa-miR-107	AC087741.1	
hsa-miR-107	LINC01970	
hsa-miR-107	AP005328.1	
hsa-miR-107	AP001094.2	
hsa-miR-107	AP000864.1	
hsa-miR-107	AC090772.3	
hsa-miR-107	AC090403.1	
hsa-miR-107	AC012123.1	
hsa-miR-107	AC090220.1	
hsa-miR-107	AC023043.4	
hsa-miR-107	AC027097.2	
hsa-miR-107	AC090236.2	
hsa-miR-107	AC125437.2	
hsa-miR-107	AC139100.2	
hsa-miR-107	AC006538.3	
hsa-miR-107	AC011444.2	
hsa-miR-107	AC011471.3	
hsa-miR-107	RAB11B-AS1	
hsa-miR-107	ZNF426-DT	
hsa-miR-107	ZNF561-AS1	
hsa-miR-107	LINC00663	
hsa-miR-107	AC011467.1	
hsa-miR-107	LINC00662	
hsa-miR-107	AC006504.5	
hsa-miR-107	AC020910.4	
hsa-miR-107	LINC01801	
hsa-miR-107	AC002128.1	
hsa-miR-107	TMEM147-AS1	
hsa-miR-107	ZNF793-AS1	
hsa-miR-107	LIPE-AS1	
hsa-miR-107	AC021092.1	
hsa-miR-107	AC073548.1	
hsa-miR-107	AC010327.5	
hsa-miR-107	AC005261.5	
hsa-miR-107	AC012313.3	
hsa-miR-107	AC012313.5	
hsa-miR-107	SDCBP2-AS1	
hsa-miR-107	ZNF337-AS1	
hsa-miR-107	AL121832.3	
hsa-miR-107	AF130359.1	
hsa-miR-107	CBR3-AS1	
hsa-miR-107	TRPM2-AS	

hsa-miR-107	AC016026.1	
hsa-miR-107	AC002470.2	
hsa-miR-107	GUSBP11	
hsa-miR-107	AP000347.2	
hsa-miR-107	ADORA2A-AS1	
hsa-miR-107	MIAT	
hsa-miR-107	MIATNB	
hsa-miR-107	TTC28-AS1	
hsa-miR-107	AC003681.1	
hsa-miR-107	Z95114.4	
hsa-miR-107	AL021707.2	
hsa-miR-107	AL021707.8	
hsa-miR-107	Z95331.1	
hsa-miR-107	MIRLET7BHG	
hsa-miR-107	AL117329.1	
hsa-miR-107	AC004656.1	
hsa-miR-107	MED14OS	
hsa-miR-107	AL157700.1	
hsa-miR-107	XIST	
hsa-miR-107	FTX	
hsa-miR-107	Z83843.1	
hsa-miR-107	LINC00630	
hsa-miR-107	AL035425.3	
hsa-miR-107	MIR503HG	
hsa-miR-107	LINC00893	
hsa-miR-107	LINC00894	
hsa-miR-1269a	LINC01128	
hsa-miR-1269a	AL645608.3	
hsa-miR-1269a	AL391244.1	
hsa-miR-1269a	LINC01134	
hsa-miR-1269a	MIR34AHG	
hsa-miR-1269a	AC254633.1	
hsa-miR-1269a	AL451042.2	
hsa-miR-1269a	LINC01772	
hsa-miR-1269a	PINK1-AS	
hsa-miR-1269a	AL662907.2	
hsa-miR-1269a	KDM4A-AS1	
hsa-miR-1269a	LINC01358	
hsa-miR-1269a	LINC01781	
hsa-miR-1269a	AC092807.3	
hsa-miR-1269a	AL160006.1	
hsa-miR-1269a	SLC16A1-AS1	
hsa-miR-1269a	LIX1L-AS1	
hsa-miR-1269a	RP4-565E6.1	
hsa-miR-1269a	CADM3-AS1	
hsa-miR-1269a	AL592435.1	
hsa-miR-1269a	CRYZL2P-SEC16B	
hsa-miR-1269a	AL359853.3	
hsa-miR-1269a	MIR181A1HG	
hsa-miR-1269a	MIR29B2CHG	
hsa-miR-1269a	AL136379.1	
hsa-miR-1269a	AL359921.2	
hsa-miR-1269a	AL591623.1	
hsa-miR-1269a	AC231981.1	
hsa-miR-1269a	RN7SL832P	
hsa-miR-1269a	AL133243.3	
hsa-miR-1269a	AC007681.1	
hsa-miR-1269a	MIR4435-2HG	
hsa-miR-1269a	AC110769.1	
hsa-miR-1269a	AC073050.1	
hsa-miR-1269a	AC019080.1	
hsa-miR-1269a	AC007038.1	
hsa-miR-1269a	AC012513.3	
hsa-miR-1269a	AC012510.1	
hsa-miR-1269a	AC073052.2	
hsa-miR-1269a	AC009407.1	
hsa-miR-1269a	THUMPD3-AS1	
hsa-miR-1269a	LINC00852	
hsa-miR-1269a	AC090948.2	
hsa-miR-1269a	AC006058.1	
hsa-miR-1269a	AC124045.1	
hsa-miR-1269a	AC104447.1	
hsa-miR-1269a	RASSF1-AS1	

hsa-miR-1269a	AC012467.2	
hsa-miR-1269a	DUBR	
hsa-miR-1269a	AC112503.2	
hsa-miR-1269a	RUVBL1-AS1	
hsa-miR-1269a	AC073288.1	
hsa-miR-1269a	KCNMB2-AS1	
hsa-miR-1269a	AC139887.2	
hsa-miR-1269a	CTBP1-AS2	
hsa-miR-1269a	AC024230.1	
hsa-miR-1269a	AC107068.1	
hsa-miR-1269a	AC097478.1	
hsa-miR-1269a	AC010442.1	
hsa-miR-1269a	BASP1-AS1	
hsa-miR-1269a	AC104118.1	
hsa-miR-1269a	LINC00461	
hsa-miR-1269a	MIR3936HG	
hsa-miR-1269a	C5orf66-AS2	
hsa-miR-1269a	AC011389.1	
hsa-miR-1269a	AL035587.1	
hsa-miR-1269a	LINC00472	
hsa-miR-1269a	LINC01010	
hsa-miR-1269a	AL354892.3	
hsa-miR-1269a	AC004691.1	
hsa-miR-1269a	AC018647.2	
hsa-miR-1269a	LINC00265	
hsa-miR-1269a	AC118758.3	
hsa-miR-1269a	LINC00174	
hsa-miR-1269a	AC004491.1	
hsa-miR-1269a	MAGI2-AS3	
hsa-miR-1269a	STAG3L5P-PVRIG2P-PILRB	
hsa-miR-1269a	AC004492.1	
hsa-miR-1269a	LINC02476	
hsa-miR-1269a	AC016831.5	
hsa-miR-1269a	MKLN1-AS	
hsa-miR-1269a	AC008264.2	
hsa-miR-1269a	LINC01287	
hsa-miR-1269a	AC037459.3	
hsa-miR-1269a	HMBOX1-IT1	
hsa-miR-1269a	GASAL1	
hsa-miR-1269a	CASC19	
hsa-miR-1269a	PEG13	
hsa-miR-1269a	AC011676.5	
hsa-miR-1269a	AL589843.1	
hsa-miR-1269a	AL358074.1	
hsa-miR-1269a	AL355987.4	
hsa-miR-1269a	AL157392.3	
hsa-miR-1269a	AL359532.1	
hsa-miR-1269a	RP11-592B15.3	
hsa-miR-1269a	AL132656.3	
hsa-miR-1269a	NUTM2B-AS1	
hsa-miR-1269a	AL731569.1	
hsa-miR-1269a	AL136982.1	
hsa-miR-1269a	AGAP11	
hsa-miR-1269a	RPARP-AS1	
hsa-miR-1269a	KCNQ1OT1	
hsa-miR-1269a	LINC02547	
hsa-miR-1269a	AC090833.1	
hsa-miR-1269a	AP002748.3	
hsa-miR-1269a	AP001157.1	
hsa-miR-1269a	AP000590.1	
hsa-miR-1269a	AP002761.4	
hsa-miR-1269a	AP003119.3	
hsa-miR-1269a	AP002840.2	
hsa-miR-1269a	BACE1-AS	
hsa-miR-1269a	AP001318.2	
hsa-miR-1269a	LOH12CR2	
hsa-miR-1269a	RASSF8-AS1	
hsa-miR-1269a	AC022364.1	
hsa-miR-1269a	AC079684.1	
hsa-miR-1269a	AC008124.1	
hsa-miR-1269a	AC025031.4	
hsa-miR-1269a	AC074032.1	
hsa-miR-1269a	AC025423.4	

hsa-miR-1269a	AC090502.1	
hsa-miR-1269a	HELLPAR	
hsa-miR-1269a	NRAV	
hsa-miR-1269a	AC131212.3	
hsa-miR-1269a	AL161772.1	
hsa-miR-1269a	N4BP2L2-IT2	
hsa-miR-1269a	AL137782.1	
hsa-miR-1269a	RBM26-AS1	
hsa-miR-1269a	AL161431.1	
hsa-miR-1269a	AL442125.1	
hsa-miR-1269a	LINC00641	
hsa-miR-1269a	AL138974.1	
hsa-miR-1269a	LINC02313	
hsa-miR-1269a	LINC00648	
hsa-miR-1269a	AL627171.1	
hsa-miR-1269a	PSMA3-AS1	
hsa-miR-1269a	AL162171.3	
hsa-miR-1269a	MEG8	
hsa-miR-1269a	AL117190.1	
hsa-miR-1269a	SNHG14	
hsa-miR-1269a	PWAR5	
hsa-miR-1269a	PWAR6	
hsa-miR-1269a	ARHGAP11B	
hsa-miR-1269a	AC116158.1	
hsa-miR-1269a	AC090510.2	
hsa-miR-1269a	GABPB1-IT1	
hsa-miR-1269a	AC007950.2	
hsa-miR-1269a	AC116913.1	
hsa-miR-1269a	AC110048.2	
hsa-miR-1269a	AC090826.1	
hsa-miR-1269a	LINC01578	
hsa-miR-1269a	AC022819.1	
hsa-miR-1269a	Z92544.2	
hsa-miR-1269a	ERVK13-1	
hsa-miR-1269a	AC092117.2	
hsa-miR-1269a	SRRM2-AS1	
hsa-miR-1269a	MIR762HG	
hsa-miR-1269a	FBXL19-AS1	
hsa-miR-1269a	AC026471.4	
hsa-miR-1269a	AC009102.2	
hsa-miR-1269a	AC025287.1	
hsa-miR-1269a	AC092139.3	
hsa-miR-1269a	AC092135.1	
hsa-miR-1269a	AC009063.2	
hsa-miR-1269a	MYHAS	
hsa-miR-1269a	AC126365.1	
hsa-miR-1269a	AC127024.8	
hsa-miR-1269a	AC004585.1	
hsa-miR-1269a	AC067852.3	
hsa-miR-1269a	ASB16-AS1	
hsa-miR-1269a	MAPT-IT1	
hsa-miR-1269a	AC007485.2	
hsa-miR-1269a	CTC-462L7.1	
hsa-miR-1269a	AC015813.6	
hsa-miR-1269a	AC005332.7	
hsa-miR-1269a	TEN1-CDK3	
hsa-miR-1269a	SNHG16	
hsa-miR-1269a	AC111170.2	
hsa-miR-1269a	AC110285.1	
hsa-miR-1269a	AC139530.3	
hsa-miR-1269a	AC145207.5	
hsa-miR-1269a	AC132872.4	
hsa-miR-1269a	AC132872.1	
hsa-miR-1269a	AP005057.1	
hsa-miR-1269a	AP001793.1	
hsa-miR-1269a	AP005131.6	
hsa-miR-1269a	AC090241.3	
hsa-miR-1269a	LINC01415	
hsa-miR-1269a	AC036176.1	
hsa-miR-1269a	AC093330.2	
hsa-miR-1269a	AC114271.1	
hsa-miR-1269a	ZNF433-AS1	
hsa-miR-1269a	AC011446.2	

hsa-miR-1269a	AC092329.4	
hsa-miR-1269a	LINC01224	
hsa-miR-1269a	AC092279.1	
hsa-miR-1269a	TMEM147-AS1	
hsa-miR-1269a	ZNF571-AS1	
hsa-miR-1269a	AC118344.1	
hsa-miR-1269a	AC118344.2	
hsa-miR-1269a	AC011462.2	
hsa-miR-1269a	AC010247.2	
hsa-miR-1269a	AC005261.1	
hsa-miR-1269a	AC012313.1	
hsa-miR-1269a	AC012313.5	
hsa-miR-1269a	SDCBP2-AS1	
hsa-miR-1269a	AL109976.1	
hsa-miR-1269a	AL121906.2	
hsa-miR-1269a	AL121753.2	
hsa-miR-1269a	AL391095.3	
hsa-miR-1269a	SNHG17	
hsa-miR-1269a	AL132655.1	
hsa-miR-1269a	BACH1-IT1	
hsa-miR-1269a	AC006946.2	
hsa-miR-1269a	LINC01311	
hsa-miR-1269a	AC002470.2	
hsa-miR-1269a	GUSBP11	
hsa-miR-1269a	LINC01422	
hsa-miR-1269a	AL022313.4	
hsa-miR-1269a	AL021707.3	
hsa-miR-1269a	Z95331.1	
hsa-miR-1269a	MIRLET7BHG	
hsa-miR-1269a	AL117329.1	
hsa-miR-1269a	BX890604.1	
hsa-miR-1269a	FAM239B	
hsa-miR-1269a	AC073529.1	
hsa-miR-1269a	AC004656.1	
hsa-miR-1269a	LINC01278	
hsa-miR-1269a	XIST	
hsa-miR-320b	AL391244.1	
hsa-miR-320b	MIR34AHG	
hsa-miR-320b	BX284668.2	
hsa-miR-320b	SNHG12	
hsa-miR-320b	AL139260.2	
hsa-miR-320b	AL031985.3	
hsa-miR-320b	LINC01748	
hsa-miR-320b	MIR137HG	
hsa-miR-320b	AL049557.1	
hsa-miR-320b	SLC16A1-AS1	
hsa-miR-320b	AC239809.3	
hsa-miR-320b	LINC01138	
hsa-miR-320b	AL590133.2	
hsa-miR-320b	AL162258.1	
hsa-miR-320b	AL590666.1	
hsa-miR-320b	GAS5	
hsa-miR-320b	AL359265.3	
hsa-miR-320b	AL356273.3	
hsa-miR-320b	AL358473.1	
hsa-miR-320b	MIR29B2CHG	
hsa-miR-320b	AL592402.1	
hsa-miR-320b	AC011700.1	
hsa-miR-320b	AL365184.1	
hsa-miR-320b	LINC01341	
hsa-miR-320b	AC141930.2	
hsa-miR-320b	LINC01814	
hsa-miR-320b	AC104794.3	
hsa-miR-320b	C2orf48	
hsa-miR-320b	AC092687.3	
hsa-miR-320b	AL133243.2	
hsa-miR-320b	LINC02576	
hsa-miR-320b	LINC01829	
hsa-miR-320b	ALMS1-IT1	
hsa-miR-320b	BOLA3-AS1	
hsa-miR-320b	AC005034.3	
hsa-miR-320b	AC233266.2	
hsa-miR-320b	LINC00342	

hsa-miR-320b	LINC01885	
hsa-miR-320b	MIR4435-2HG	
hsa-miR-320b	AC079753.1	
hsa-miR-320b	PAX8-AS1	
hsa-miR-320b	AC009299.3	
hsa-miR-320b	AC007405.1	
hsa-miR-320b	AC007405.3	
hsa-miR-320b	AC019080.1	
hsa-miR-320b	AC012499.1	
hsa-miR-320b	AC009948.1	
hsa-miR-320b	TTN-AS1	
hsa-miR-320b	AC009962.1	
hsa-miR-320b	AC007966.1	
hsa-miR-320b	AC017101.1	
hsa-miR-320b	AC007383.3	
hsa-miR-320b	LINC01963	
hsa-miR-320b	AC012510.1	
hsa-miR-320b	AC067956.1	
hsa-miR-320b	THUMPD3-AS1	
hsa-miR-320b	GHRLOS	
hsa-miR-320b	FGD5-AS1	
hsa-miR-320b	SH3BP5-AS1	
hsa-miR-320b	KIF9-AS1	
hsa-miR-320b	ZMYND10-AS1	
hsa-miR-320b	AC073352.1	
hsa-miR-320b	AC022336.3	
hsa-miR-320b	AC092902.2	
hsa-miR-320b	LINC01471	
hsa-miR-320b	MBNL1-AS1	
hsa-miR-320b	KCNMB2-AS1	
hsa-miR-320b	SOX2-OT	
hsa-miR-320b	MCCC1-AS1	
hsa-miR-320b	AC005674.2	
hsa-miR-320b	AL136537.2	
hsa-miR-320b	AC093725.1	
hsa-miR-320b	AC124016.1	
hsa-miR-320b	THAP9-AS1	
hsa-miR-320b	AP002026.1	
hsa-miR-320b	RPL34-AS1	
hsa-miR-320b	AC093908.1	
hsa-miR-320b	MIR4453HG	
hsa-miR-320b	AC104083.1	
hsa-miR-320b	HAND2-AS1	
hsa-miR-320b	AC093909.6	
hsa-miR-320b	AC021087.3	
hsa-miR-320b	MIR4458HG	
hsa-miR-320b	LINC01194	
hsa-miR-320b	AC139792.3	
hsa-miR-320b	AC025171.2	
hsa-miR-320b	NNT-AS1	
hsa-miR-320b	AC010478.1	
hsa-miR-320b	AC024581.1	
hsa-miR-320b	LUCAT1	
hsa-miR-320b	NR2F1-AS1	
hsa-miR-320b	AC099520.1	
hsa-miR-320b	LINC00992	
hsa-miR-320b	AC008669.1	
hsa-miR-320b	LINC01184	
hsa-miR-320b	AC244517.1	
hsa-miR-320b	AC021078.1	
hsa-miR-320b	MIR3142HG	
hsa-miR-320b	AC022217.3	
hsa-miR-320b	ZNF346-IT1	
hsa-miR-320b	GMDS-AS1	
hsa-miR-320b	AL031963.1	
hsa-miR-320b	AL359643.3	
hsa-miR-320b	AL031775.1	
hsa-miR-320b	Z97832.2	
hsa-miR-320b	AL512274.1	
hsa-miR-320b	AL096865.1	
hsa-miR-320b	TRAM2-AS1	
hsa-miR-320b	AL049555.1	
hsa-miR-320b	AL136164.3	

hsa-miR-320b	LINC00472	
hsa-miR-320b	MANEA-AS1	
hsa-miR-320b	AL513550.1	
hsa-miR-320b	LINC02532	
hsa-miR-320b	TRAF3IP2-AS1	
hsa-miR-320b	AL356124.1	
hsa-miR-320b	AL031320.2	
hsa-miR-320b	AL080276.2	
hsa-miR-320b	LINC00473	
hsa-miR-320b	AC091729.3	
hsa-miR-320b	AC006041.1	
hsa-miR-320b	AC005154.1	
hsa-miR-320b	LINC00997	
hsa-miR-320b	TRG-AS1	
hsa-miR-320b	AC017116.1	
hsa-miR-320b	AC118758.3	
hsa-miR-320b	LINC00174	
hsa-miR-320b	AC073335.2	
hsa-miR-320b	APTR	
hsa-miR-320b	MAGI2-AS3	
hsa-miR-320b	AC000120.1	
hsa-miR-320b	STAG3L5P-PVRIG2P-PILRB	
hsa-miR-320b	AC069281.2	
hsa-miR-320b	ST7-OT4	
hsa-miR-320b	SND1-IT1	
hsa-miR-320b	AC090114.2	
hsa-miR-320b	AC016831.1	
hsa-miR-320b	AC016831.7	
hsa-miR-320b	ATP6V0E2-AS1	
hsa-miR-320b	AC144652.1	
hsa-miR-320b	LINC00689	
hsa-miR-320b	AC016065.1	
hsa-miR-320b	AF131216.3	
hsa-miR-320b	EXTL3-AS1	
hsa-miR-320b	AC102945.2	
hsa-miR-320b	AC090152.1	
hsa-miR-320b	MIR124-2HG	
hsa-miR-320b	AC018616.1	
hsa-miR-320b	LINC01419	
hsa-miR-320b	AC090198.1	
hsa-miR-320b	PEG13	
hsa-miR-320b	CDC37L1-AS1	
hsa-miR-320b	FAM201A	
hsa-miR-320b	AL158152.1	
hsa-miR-320b	NAMA	
hsa-miR-320b	FAM225A	
hsa-miR-320b	AL162724.1	
hsa-miR-320b	AL441992.1	
hsa-miR-320b	LINC00963	
hsa-miR-320b	DBH-AS1	
hsa-miR-320b	CCDC183-AS1	
hsa-miR-320b	AL355987.4	
hsa-miR-320b	AL390294.1	
hsa-miR-320b	AC073367.1	
hsa-miR-320b	AL359697.1	
hsa-miR-320b	WAC-AS1	
hsa-miR-320b	AL353796.1	
hsa-miR-320b	AC022400.3	
hsa-miR-320b	BMS1P4	
hsa-miR-320b	ZNF503-AS2	
hsa-miR-320b	AC010997.6	
hsa-miR-320b	NUTM2B-AS1	
hsa-miR-320b	AL158212.3	
hsa-miR-320b	AL391988.1	
hsa-miR-320b	AL139407.1	
hsa-miR-320b	AL162274.3	
hsa-miR-320b	AC124057.1	
hsa-miR-320b	KCNQ1OT1	
hsa-miR-320b	AC022762.2	
hsa-miR-320b	MRVI1-AS1	
hsa-miR-320b	LINC00294	
hsa-miR-320b	SNHG1	
hsa-miR-320b	NEAT1	

hsa-miR-320b	MALAT1	
hsa-miR-320b	AP000808.1	
hsa-miR-320b	AP002490.1	
hsa-miR-320b	AP001922.6	
hsa-miR-320b	AP002360.1	
hsa-miR-320b	AP000941.1	
hsa-miR-320b	LINC00167	
hsa-miR-320b	AC007406.5	
hsa-miR-320b	AC005912.2	
hsa-miR-320b	AC010186.3	
hsa-miR-320b	AC008124.1	
hsa-miR-320b	AC025031.5	
hsa-miR-320b	AC004466.2	
hsa-miR-320b	AC023509.1	
hsa-miR-320b	AC012531.1	
hsa-miR-320b	AC023794.7	
hsa-miR-320b	AC026124.1	
hsa-miR-320b	AC090061.1	
hsa-miR-320b	AC020656.2	
hsa-miR-320b	TMPO-AS1	
hsa-miR-320b	HELLPAR	
hsa-miR-320b	AC144548.1	
hsa-miR-320b	PXN-AS1	
hsa-miR-320b	AC069234.3	
hsa-miR-320b	HNF1A-AS1	
hsa-miR-320b	LINC01089	
hsa-miR-320b	AC027290.2	
hsa-miR-320b	AC068790.3	
hsa-miR-320b	AC073592.7	
hsa-miR-320b	AL161772.1	
hsa-miR-320b	AL138820.1	
hsa-miR-320b	AL138999.1	
hsa-miR-320b	TPT1-AS1	
hsa-miR-320b	LINC00562	
hsa-miR-320b	DLEU1	
hsa-miR-320b	RNASEH2B-AS1	
hsa-miR-320b	AL137782.1	
hsa-miR-320b	RBM26-AS1	
hsa-miR-320b	LINC00346	
hsa-miR-320b	AL442125.1	
hsa-miR-320b	LINC00641	
hsa-miR-320b	AL132780.4	
hsa-miR-320b	ZFHX2-AS1	
hsa-miR-320b	DHRS4-AS1	
hsa-miR-320b	AL162311.3	
hsa-miR-320b	LINC00648	
hsa-miR-320b	FRMD6-AS1	
hsa-miR-320b	PSMA3-AS1	
hsa-miR-320b	LINC01303	
hsa-miR-320b	AC004846.2	
hsa-miR-320b	AC016526.3	
hsa-miR-320b	AL049775.1	
hsa-miR-320b	MEG3	
hsa-miR-320b	SNHG14	
hsa-miR-320b	AC124303.1	
hsa-miR-320b	AC019278.1	
hsa-miR-320b	AC113146.1	
hsa-miR-320b	RAD51-AS1	
hsa-miR-320b	OIP5-AS1	
hsa-miR-320b	GABPB1-IT1	
hsa-miR-320b	DNAAF4-CCPG1	
hsa-miR-320b	AC011939.2	
hsa-miR-320b	AC116913.1	
hsa-miR-320b	AC100827.4	
hsa-miR-320b	NPTN-IT1	
hsa-miR-320b	AC090826.2	
hsa-miR-320b	AC068338.2	
hsa-miR-320b	AC090181.2	
hsa-miR-320b	AC021739.3	
hsa-miR-320b	MIR9-3HG	
hsa-miR-320b	ZNF710-AS1	
hsa-miR-320b	NR2F2-AS1	
hsa-miR-320b	AC015712.2	

hsa-miR-320b	Z92544.2	
hsa-miR-320b	AL133297.2	
hsa-miR-320b	ERVK13-1	
hsa-miR-320b	MMP25-AS1	
hsa-miR-320b	ZNF213-AS1	
hsa-miR-320b	AC131649.2	
hsa-miR-320b	AC007216.4	
hsa-miR-320b	AC127459.3	
hsa-miR-320b	AC008741.2	
hsa-miR-320b	RRN3P2	
hsa-miR-320b	AC009133.1	
hsa-miR-320b	AC120114.2	
hsa-miR-320b	AC007490.1	
hsa-miR-320b	AC020978.7	
hsa-miR-320b	AC009032.1	
hsa-miR-320b	LINC01572	
hsa-miR-320b	AC004943.2	
hsa-miR-320b	AC002044.1	
hsa-miR-320b	AC009120.2	
hsa-miR-320b	AC092718.4	
hsa-miR-320b	AC113189.4	
hsa-miR-320b	AC016876.2	
hsa-miR-320b	AC233702.9	
hsa-miR-320b	AC005899.8	
hsa-miR-320b	AC004408.2	
hsa-miR-320b	AC099811.1	
hsa-miR-320b	AC099811.5	
hsa-miR-320b	AC067852.1	
hsa-miR-320b	LINC00910	
hsa-miR-320b	ASB16-AS1	
hsa-miR-320b	MAPT-IT1	
hsa-miR-320b	AC103702.1	
hsa-miR-320b	AC015909.5	
hsa-miR-320b	AC007485.2	
hsa-miR-320b	AC091181.1	
hsa-miR-320b	AC015813.1	
hsa-miR-320b	AC015813.6	
hsa-miR-320b	AC091271.1	
hsa-miR-320b	AC005746.1	
hsa-miR-320b	AC005821.1	
hsa-miR-320b	AC068512.1	
hsa-miR-320b	AC005332.7	
hsa-miR-320b	SOX9-AS1	
hsa-miR-320b	AC111170.3	
hsa-miR-320b	BAIAP2-AS1	
hsa-miR-320b	AC145207.5	
hsa-miR-320b	AP001005.3	
hsa-miR-320b	AP000845.1	
hsa-miR-320b	TYMSOS	
hsa-miR-320b	AP000894.4	
hsa-miR-320b	DLGAP1-AS2	
hsa-miR-320b	LINC00667	
hsa-miR-320b	AP005432.1	
hsa-miR-320b	AP005131.7	
hsa-miR-320b	AC105114.1	
hsa-miR-320b	AC007996.1	
hsa-miR-320b	AC023043.4	
hsa-miR-320b	AC022031.1	
hsa-miR-320b	AC105105.2	
hsa-miR-320b	LINC00909	
hsa-miR-320b	AC134978.1	
hsa-miR-320b	RBFADN	
hsa-miR-320b	AC136469.2	
hsa-miR-320b	ILF3-AS1	
hsa-miR-320b	AC020916.1	
hsa-miR-320b	AC003956.1	
hsa-miR-320b	AC020917.4	
hsa-miR-320b	LINC00663	
hsa-miR-320b	AC092279.2	
hsa-miR-320b	TMEM147-AS1	
hsa-miR-320b	LIPE-AS1	
hsa-miR-320b	AC007191.1	
hsa-miR-320b	AC008895.1	

hsa-miR-320b	AC008403.3	
hsa-miR-320b	AC022150.4	
hsa-miR-320b	AC004076.2	
hsa-miR-320b	AC012313.8	
hsa-miR-320b	SDCBP2-AS1	
hsa-miR-320b	CASC20	
hsa-miR-320b	AL161658.1	
hsa-miR-320b	LINC00261	
hsa-miR-320b	AL031673.1	
hsa-miR-320b	AL121832.3	
hsa-miR-320b	AL118506.1	
hsa-miR-320b	AP000251.1	
hsa-miR-320b	LINC01547	
hsa-miR-320b	MCM3AP-AS1	
hsa-miR-320b	AC016026.1	
hsa-miR-320b	GUSBP11	
hsa-miR-320b	AP000355.1	
hsa-miR-320b	AL008721.2	
hsa-miR-320b	MIAT	
hsa-miR-320b	LINC01638	
hsa-miR-320b	TTC28-AS1	
hsa-miR-320b	AC003681.1	
hsa-miR-320b	TUG1	
hsa-miR-320b	LINC01521	
hsa-miR-320b	AL021877.2	
hsa-miR-320b	AL022311.1	
hsa-miR-320b	AL021707.1	
hsa-miR-320b	AL031595.3	
hsa-miR-320b	MIRLET7BHG	
hsa-miR-320b	AC004656.1	
hsa-miR-320b	MIR222HG	
hsa-miR-320b	XIST	
hsa-miR-320b	FTX	
hsa-miR-320b	AL035425.4	
hsa-miR-320b	AC244090.3	
hsa-miR-320b	TTTY15	
hsa-miR-326	AL645608.3	
hsa-miR-326	AL645608.1	
hsa-miR-326	AL645608.9	
hsa-miR-326	AL162741.1	
hsa-miR-326	AL391244.1	
hsa-miR-326	AL691432.2	
hsa-miR-326	AL031282.2	
hsa-miR-326	LINC00982	
hsa-miR-326	AL512383.1	
hsa-miR-326	AL513320.1	
hsa-miR-326	LINC01134	
hsa-miR-326	BX284668.5	
hsa-miR-326	PINK1-AS	
hsa-miR-326	LINC01355	
hsa-miR-326	AL021154.1	
hsa-miR-326	SNHG3	
hsa-miR-326	SNHG12	
hsa-miR-326	AL662907.1	
hsa-miR-326	AL662907.2	
hsa-miR-326	AL513327.1	
hsa-miR-326	AL139260.1	
hsa-miR-326	SLFNL1-AS1	
hsa-miR-326	KDM4A-AS1	
hsa-miR-326	FOXD2-AS1	
hsa-miR-326	AL355483.1	
hsa-miR-326	AL357673.2	
hsa-miR-326	LINC01748	
hsa-miR-326	LINC01359	
hsa-miR-326	AL590094.1	
hsa-miR-326	AC095033.1	
hsa-miR-326	MIR137HG	
hsa-miR-326	AL359258.2	
hsa-miR-326	AL359258.3	
hsa-miR-326	AL356488.3	
hsa-miR-326	AL160006.1	
hsa-miR-326	SLC16A1-AS1	
hsa-miR-326	AL359915.2	

hsa-miR-326	RP6-42F4.1	
hsa-miR-326	LIX1L-AS1	
hsa-miR-326	AC239809.3	
hsa-miR-326	RP4-565E6.1	
hsa-miR-326	XXyac-YX155B6.6	
hsa-miR-326	ADAMTSL4-AS1	
hsa-miR-326	AL162258.1	
hsa-miR-326	AL162258.2	
hsa-miR-326	RUSC1-AS1	
hsa-miR-326	AL365181.2	
hsa-miR-326	AL365181.3	
hsa-miR-326	KIRREL1-IT1	
hsa-miR-326	AL031733.2	
hsa-miR-326	AL353708.1	
hsa-miR-326	AL162431.2	
hsa-miR-326	PCAT6	
hsa-miR-326	AL445493.2	
hsa-miR-326	MIR29B2CHG	
hsa-miR-326	PROX1-AS1	
hsa-miR-326	AC093152.1	
hsa-miR-326	AL670729.1	
hsa-miR-326	AL359921.2	
hsa-miR-326	AL359924.1	
hsa-miR-326	C2orf48	
hsa-miR-326	RN7SL832P	
hsa-miR-326	AC104695.4	
hsa-miR-326	AC016747.4	
hsa-miR-326	PCBP1-AS1	
hsa-miR-326	LINC01159	
hsa-miR-326	MIR4435-2HG	
hsa-miR-326	AC079753.1	
hsa-miR-326	PAX8-AS1	
hsa-miR-326	AC114763.1	
hsa-miR-326	LINC01876	
hsa-miR-326	HAGLR	
hsa-miR-326	HAGLROS	
hsa-miR-326	AC007383.4	
hsa-miR-326	AC007038.1	
hsa-miR-326	AC012513.3	
hsa-miR-326	AC005538.2	
hsa-miR-326	AC012485.1	
hsa-miR-326	AC110619.1	
hsa-miR-326	GHRLOS	
hsa-miR-326	AC097639.1	
hsa-miR-326	EIF1B-AS1	
hsa-miR-326	KIF9-AS1	
hsa-miR-326	U73166.1	
hsa-miR-326	Z84492.1	
hsa-miR-326	AC115284.1	
hsa-miR-326	AC096887.2	
hsa-miR-326	PSMD6-AS2	
hsa-miR-326	FOXP1-AS1	
hsa-miR-326	LINC00960	
hsa-miR-326	LINC02035	
hsa-miR-326	AC022336.3	
hsa-miR-326	AC112484.3	
hsa-miR-326	H1FX-AS1	
hsa-miR-326	LINC02004	
hsa-miR-326	AC097103.2	
hsa-miR-326	AC021074.3	
hsa-miR-326	AC107021.2	
hsa-miR-326	ARHGEF26-AS1	
hsa-miR-326	LINC02043	
hsa-miR-326	LPP-AS2	
hsa-miR-326	NOP14-AS1	
hsa-miR-326	AFAP1-AS1	
hsa-miR-326	AC005674.2	
hsa-miR-326	CPEB2-AS1	
hsa-miR-326	TAPT1-AS1	
hsa-miR-326	AC107398.3	
hsa-miR-326	AP002026.1	
hsa-miR-326	AC108062.1	
hsa-miR-326	AC097376.2	

hsa-miR-326	HAND2-AS1	
hsa-miR-326	AC097652.1	
hsa-miR-326	EXOC3-AS1	
hsa-miR-326	AC026740.1	
hsa-miR-326	AC034229.2	
hsa-miR-326	AC034229.3	
hsa-miR-326	AC008957.3	
hsa-miR-326	AC025171.2	
hsa-miR-326	AC008966.1	
hsa-miR-326	AC010359.1	
hsa-miR-326	AC093535.2	
hsa-miR-326	C5orf66	
hsa-miR-326	CXXC5-AS1	
hsa-miR-326	CARMN	
hsa-miR-326	AC008443.1	
hsa-miR-326	GMDS-AS1	
hsa-miR-326	LINC01011	
hsa-miR-326	AL590004.4	
hsa-miR-326	HCP5	
hsa-miR-326	AL592158.1	
hsa-miR-326	AL512274.1	
hsa-miR-326	AL354719.2	
hsa-miR-326	AL160262.1	
hsa-miR-326	LINC00472	
hsa-miR-326	AC019205.1	
hsa-miR-326	AL359715.3	
hsa-miR-326	AL023284.4	
hsa-miR-326	LINC00473	
hsa-miR-326	PSMG3-AS1	
hsa-miR-326	AC092171.5	
hsa-miR-326	AC092171.2	
hsa-miR-326	RNF216-IT1	
hsa-miR-326	POLR2J4	
hsa-miR-326	AC004951.1	
hsa-miR-326	AC017116.1	
hsa-miR-326	AC073335.2	
hsa-miR-326	AC005089.1	
hsa-miR-326	STAG3L5P-PVRIG2P-PILRB	
hsa-miR-326	AC069281.2	
hsa-miR-326	AC005046.1	
hsa-miR-326	POT1-AS1	
hsa-miR-326	SND1-IT1	
hsa-miR-326	LINC-PINT	
hsa-miR-326	AC010973.2	
hsa-miR-326	LINC01287	
hsa-miR-326	AC006372.3	
hsa-miR-326	AF131215.5	
hsa-miR-326	AC015468.4	
hsa-miR-326	AC055854.1	
hsa-miR-326	AC104958.2	
hsa-miR-326	PCAT1	
hsa-miR-326	PVT1	
hsa-miR-326	PEG13	
hsa-miR-326	LY6E-DT	
hsa-miR-326	VLDLR-AS1	
hsa-miR-326	LINC01242	
hsa-miR-326	AL353795.3	
hsa-miR-326	AL590705.5	
hsa-miR-326	AL138756.1	
hsa-miR-326	GSN-AS1	
hsa-miR-326	MIR600HG	
hsa-miR-326	AL162724.1	
hsa-miR-326	C9orf106	
hsa-miR-326	LINC01503	
hsa-miR-326	LINC00963	
hsa-miR-326	BX649632.1	
hsa-miR-326	AL138781.1	
hsa-miR-326	SNHG7	
hsa-miR-326	CCDC183-AS1	
hsa-miR-326	AL355987.4	
hsa-miR-326	STPG3-AS1	
hsa-miR-326	ARRDC1-AS1	
hsa-miR-326	AL137145.1	

hsa-miR-326	AL157392.3	
hsa-miR-326	ZEB1-AS1	
hsa-miR-326	TMEM72-AS1	
hsa-miR-326	AL513534.1	
hsa-miR-326	AC022400.7	
hsa-miR-326	LINC00857	
hsa-miR-326	AL365434.1	
hsa-miR-326	ENTPD1-AS1	
hsa-miR-326	RPARP-AS1	
hsa-miR-326	AL158212.3	
hsa-miR-326	AL390763.2	
hsa-miR-326	AC069287.2	
hsa-miR-326	AP006284.1	
hsa-miR-326	H19	
hsa-miR-326	AC124057.1	
hsa-miR-326	KCNQ1OT1	
hsa-miR-326	BDNF-AS	
hsa-miR-326	WT1-AS	
hsa-miR-326	AL136146.2	
hsa-miR-326	AC129502.1	
hsa-miR-326	AC116021.1	
hsa-miR-326	SNHG1	
hsa-miR-326	NEAT1	
hsa-miR-326	MALAT1	
hsa-miR-326	AP000879.2	
hsa-miR-326	AP002336.1	
hsa-miR-326	LINC01537	
hsa-miR-326	AP001972.5	
hsa-miR-326	AP001922.6	
hsa-miR-326	COLCA1	
hsa-miR-326	AP000757.1	
hsa-miR-326	AP003486.1	
hsa-miR-326	C1RL-AS1	
hsa-miR-326	FAM66C	
hsa-miR-326	AC010186.3	
hsa-miR-326	LINC00941	
hsa-miR-326	AC025259.1	
hsa-miR-326	AC068888.1	
hsa-miR-326	HOTAIR	
hsa-miR-326	HOXC-AS3	
hsa-miR-326	SMUG1-AS1	
hsa-miR-326	AC023794.7	
hsa-miR-326	AC025165.1	
hsa-miR-326	AC084033.3	
hsa-miR-326	AC090502.1	
hsa-miR-326	LINC02407	
hsa-miR-326	HELLPAR	
hsa-miR-326	AC007637.1	
hsa-miR-326	AC144548.1	
hsa-miR-326	NRAV	
hsa-miR-326	AC026362.1	
hsa-miR-326	AC026362.2	
hsa-miR-326	AC055713.1	
hsa-miR-326	AC068790.9	
hsa-miR-326	AC122688.3	
hsa-miR-326	FZD10-AS1	
hsa-miR-326	AC131212.3	
hsa-miR-326	AC131212.2	
hsa-miR-326	PCOTH	
hsa-miR-326	LINC00412	
hsa-miR-326	AL161891.1	
hsa-miR-326	DLEU2	
hsa-miR-326	GAS6-AS1	
hsa-miR-326	AL355075.4	
hsa-miR-326	AL132780.2	
hsa-miR-326	AL132780.4	
hsa-miR-326	ZFHX2-AS1	
hsa-miR-326	AL135999.1	
hsa-miR-326	DHRS4-AS1	
hsa-miR-326	AL096870.2	
hsa-miR-326	AL133372.2	
hsa-miR-326	G2E3-AS1	
hsa-miR-326	LINC02313	

hsa-miR-326	AL158801.2	
hsa-miR-326	LINC01303	
hsa-miR-326	FUT8-AS1	
hsa-miR-326	LINC01220	
hsa-miR-326	VASH1-AS1	
hsa-miR-326	AL355838.1	
hsa-miR-326	AL162171.3	
hsa-miR-326	AL137786.1	
hsa-miR-326	AL049833.3	
hsa-miR-326	MEG3	
hsa-miR-326	DIO3OS	
hsa-miR-326	AL583722.2	
hsa-miR-326	SNHG14	
hsa-miR-326	AC124312.3	
hsa-miR-326	AC127502.2	
hsa-miR-326	AC013652.1	
hsa-miR-326	OIP5-AS1	
hsa-miR-326	GABPB1-AS1	
hsa-miR-326	AC020891.2	
hsa-miR-326	AC011912.1	
hsa-miR-326	AC010999.3	
hsa-miR-326	AC018904.2	
hsa-miR-326	AC007950.2	
hsa-miR-326	AC110048.2	
hsa-miR-326	AC026992.2	
hsa-miR-326	AC009690.2	
hsa-miR-326	TMEM202-AS1	
hsa-miR-326	AC100827.4	
hsa-miR-326	NPTN-IT1	
hsa-miR-326	DNM1P35	
hsa-miR-326	TMC3-AS1	
hsa-miR-326	MIR9-3HG	
hsa-miR-326	ZNF710-AS1	
hsa-miR-326	NR2F2-AS1	
hsa-miR-326	AC036108.2	
hsa-miR-326	SNHG9	
hsa-miR-326	AC106820.4	
hsa-miR-326	AC093525.10	
hsa-miR-326	AC141586.4	
hsa-miR-326	AC092117.1	
hsa-miR-326	MMP25-AS1	
hsa-miR-326	AC022167.2	
hsa-miR-326	AC009133.1	
hsa-miR-326	SLX1A-SULT1A3	
hsa-miR-326	AC106782.6	
hsa-miR-326	AC026471.1	
hsa-miR-326	AC026471.6	
hsa-miR-326	AC009061.2	
hsa-miR-326	AC040162.3	
hsa-miR-326	AC020978.1	
hsa-miR-326	AC126773.2	
hsa-miR-326	AC009022.1	
hsa-miR-326	AC020763.4	
hsa-miR-326	AC009078.3	
hsa-miR-326	AC136603.1	
hsa-miR-326	AC092135.3	
hsa-miR-326	AC092127.1	
hsa-miR-326	AC105429.1	
hsa-miR-326	SNAI3-AS1	
hsa-miR-326	AC092384.3	
hsa-miR-326	AC099684.2	
hsa-miR-326	AC005696.4	
hsa-miR-326	AC004771.2	
hsa-miR-326	AC113189.2	
hsa-miR-326	AC113189.4	
hsa-miR-326	AC016876.2	
hsa-miR-326	AC005224.4	
hsa-miR-326	LRRC75A-AS1	
hsa-miR-326	AC055811.4	
hsa-miR-326	AC087163.3	
hsa-miR-326	AC026271.3	
hsa-miR-326	CCDC144NL-AS1	
hsa-miR-326	AC233702.9	

hsa-miR-326	AC002094.1	
hsa-miR-326	AC010761.1	
hsa-miR-326	AC011840.6	
hsa-miR-326	AC018629.1	
hsa-miR-326	AC125257.1	
hsa-miR-326	AC099811.1	
hsa-miR-326	AC067852.2	
hsa-miR-326	AC060780.1	
hsa-miR-326	AC003102.1	
hsa-miR-326	AC142472.1	
hsa-miR-326	AC008105.3	
hsa-miR-326	AC018521.1	
hsa-miR-326	LINC02086	
hsa-miR-326	AC015909.5	
hsa-miR-326	AC005921.3	
hsa-miR-326	TOB1-AS1	
hsa-miR-326	AC004584.3	
hsa-miR-326	AC091181.1	
hsa-miR-326	AC015883.1	
hsa-miR-326	AC007431.3	
hsa-miR-326	AC015813.1	
hsa-miR-326	ARHGAP27P1-BPTFP1-KPNA2P3	
hsa-miR-326	AC005332.6	
hsa-miR-326	LINC01482	
hsa-miR-326	AC016888.1	
hsa-miR-326	AC022211.2	
hsa-miR-326	TEN1-CDK3	
hsa-miR-326	SNHG16	
hsa-miR-326	SNHG20	
hsa-miR-326	AC100788.2	
hsa-miR-326	AC087741.1	
hsa-miR-326	AC124319.2	
hsa-miR-326	AC120024.4	
hsa-miR-326	LINC00482	
hsa-miR-326	AC110285.6	
hsa-miR-326	AC139149.1	
hsa-miR-326	AC132872.3	
hsa-miR-326	HEXDC-IT1	
hsa-miR-326	AC124283.5	
hsa-miR-326	LINC00667	
hsa-miR-326	NDUFV2-AS1	
hsa-miR-326	AP001269.4	
hsa-miR-326	AP005131.6	
hsa-miR-326	AC090220.1	
hsa-miR-326	AC022031.2	
hsa-miR-326	AC105105.2	
hsa-miR-326	AC009716.2	
hsa-miR-326	AC093330.1	
hsa-miR-326	AC093330.2	
hsa-miR-326	AC134978.1	
hsa-miR-326	AC009005.1	
hsa-miR-326	AC005329.3	
hsa-miR-326	AC027307.2	
hsa-miR-326	CACTIN-AS1	
hsa-miR-326	AC011498.7	
hsa-miR-326	AC011491.2	
hsa-miR-326	AC010336.1	
hsa-miR-326	AC010336.4	
hsa-miR-326	AC020917.2	
hsa-miR-326	AC008764.10	
hsa-miR-326	AC024075.2	
hsa-miR-326	AC138430.1	
hsa-miR-326	LINC00663	
hsa-miR-326	AC092329.4	
hsa-miR-326	AC007786.3	
hsa-miR-326	LINC01834	
hsa-miR-326	AC008738.7	
hsa-miR-326	CEBPA-AS1	
hsa-miR-326	TMEM147-AS1	
hsa-miR-326	AC002116.1	
hsa-miR-326	LINC00665	
hsa-miR-326	AC022144.1	
hsa-miR-326	AC118344.2	

hsa-miR-326	LIPE-AS1	
hsa-miR-326	AC243964.3	
hsa-miR-326	AC008895.1	
hsa-miR-326	AC022150.4	
hsa-miR-326	AC010327.3	
hsa-miR-326	AC010327.4	
hsa-miR-326	AC010327.5	
hsa-miR-326	ZNF460-AS1	
hsa-miR-326	MZF1-AS1	
hsa-miR-326	AL117335.1	
hsa-miR-326	AL121761.2	
hsa-miR-326	LINC00261	
hsa-miR-326	ZNF337-AS1	
hsa-miR-326	AL391095.1	
hsa-miR-326	LINC01270	
hsa-miR-326	AL121832.3	
hsa-miR-326	AL121829.2	
hsa-miR-326	LINC01666	
hsa-miR-326	CR381653.1	
hsa-miR-326	MIR548XHG	
hsa-miR-326	LINC00649	
hsa-miR-326	BACE2-IT1	
hsa-miR-326	PLAC4	
hsa-miR-326	LINC01671	
hsa-miR-326	LINC00313	
hsa-miR-326	LINC01669	
hsa-miR-326	AP001062.1	
hsa-miR-326	TRPM2-AS	
hsa-miR-326	TSPEAR-AS2	
hsa-miR-326	AP001471.1	
hsa-miR-326	MCM3AP-AS1	
hsa-miR-326	CECR3	
hsa-miR-326	DGCR11	
hsa-miR-326	AC004471.1	
hsa-miR-326	AC002470.2	
hsa-miR-326	THAP7-AS1	
hsa-miR-326	AP000346.1	
hsa-miR-326	GUSBP11	
hsa-miR-326	AP000350.6	
hsa-miR-326	AL008721.2	
hsa-miR-326	Z99774.1	
hsa-miR-326	MIAT	
hsa-miR-326	TTC28-AS1	
hsa-miR-326	TUG1	
hsa-miR-326	Z94160.1	
hsa-miR-326	AL022311.1	
hsa-miR-326	AL021707.6	
hsa-miR-326	Z83851.1	
hsa-miR-326	AL022476.1	
hsa-miR-326	AL031595.3	
hsa-miR-326	BX324167.2	
hsa-miR-326	AL121672.3	
hsa-miR-326	PRR34-AS1	
hsa-miR-326	MIRLET7BHG	
hsa-miR-326	FP325330.3	
hsa-miR-326	Z97192.3	
hsa-miR-326	BX539320.1	
hsa-miR-326	AL022328.4	
hsa-miR-326	AC004656.1	
hsa-miR-326	AC231533.1	
hsa-miR-326	AC232271.1	
hsa-miR-326	XIST	
hsa-miR-326	AC234782.2	
hsa-miR-326	DANT2	
hsa-miR-326	LINC00632	
hsa-miR-326	LINC00894	
hsa-miR-326	ASMTL-AS1	
hsa-miR-326	AC011297.1	
hsa-miR-3666	AL645608.1	
hsa-miR-3666	AL391244.3	
hsa-miR-3666	AL603962.1	
hsa-miR-3666	PINK1-AS	
hsa-miR-3666	AL513327.2	

hsa-miR-3666	AL603839.3	
hsa-miR-3666	AL139289.2	
hsa-miR-3666	AC096536.1	
hsa-miR-3666	LINC01748	
hsa-miR-3666	LINC01359	
hsa-miR-3666	CCDC18-AS1	
hsa-miR-3666	SLC16A1-AS1	
hsa-miR-3666	HIPK1-AS1	
hsa-miR-3666	AL390066.1	
hsa-miR-3666	AC244021.1	
hsa-miR-3666	AC239809.3	
hsa-miR-3666	AC242426.2	
hsa-miR-3666	AC242426.3	
hsa-miR-3666	AC234582.1	
hsa-miR-3666	ASH1L-IT1	
hsa-miR-3666	KIRREL1-IT1	
hsa-miR-3666	DNM3OS	
hsa-miR-3666	MIR29B2CHG	
hsa-miR-3666	AL645465.1	
hsa-miR-3666	AC104794.2	
hsa-miR-3666	AC130710.1	
hsa-miR-3666	AC115619.1	
hsa-miR-3666	AL133243.2	
hsa-miR-3666	AC008280.3	
hsa-miR-3666	AC007744.1	
hsa-miR-3666	PCBP1-AS1	
hsa-miR-3666	BOLA3-AS1	
hsa-miR-3666	AC005034.5	
hsa-miR-3666	AC159540.1	
hsa-miR-3666	PAX8-AS1	
hsa-miR-3666	AC016710.1	
hsa-miR-3666	AC009951.1	
hsa-miR-3666	LINC01876	
hsa-miR-3666	HAGLR	
hsa-miR-3666	AC009962.1	
hsa-miR-3666	LINC01790	
hsa-miR-3666	AC064836.3	
hsa-miR-3666	AC007038.1	
hsa-miR-3666	AC021016.2	
hsa-miR-3666	AC016717.2	
hsa-miR-3666	LINC01266	
hsa-miR-3666	THUMPD3-AS1	
hsa-miR-3666	LINC00852	
hsa-miR-3666	FGD5-AS1	
hsa-miR-3666	AC090948.2	
hsa-miR-3666	AC104447.1	
hsa-miR-3666	AC012467.2	
hsa-miR-3666	AC109587.1	
hsa-miR-3666	LINC02035	
hsa-miR-3666	AC022336.3	
hsa-miR-3666	AC092902.2	
hsa-miR-3666	AC010210.1	
hsa-miR-3666	AC097103.2	
hsa-miR-3666	SOX2-OT	
hsa-miR-3666	LPP-AS2	
hsa-miR-3666	AC124944.1	
hsa-miR-3666	LINC02473	
hsa-miR-3666	LINC02484	
hsa-miR-3666	AC116049.2	
hsa-miR-3666	AC096721.1	
hsa-miR-3666	AC124016.2	
hsa-miR-3666	AC097376.2	
hsa-miR-3666	AC104083.1	
hsa-miR-3666	AC078881.1	
hsa-miR-3666	AC099343.3	
hsa-miR-3666	NNT-AS1	
hsa-miR-3666	AC022107.1	
hsa-miR-3666	AC026427.1	
hsa-miR-3666	CKMT2-AS1	
hsa-miR-3666	TMEM161B-AS1	
hsa-miR-3666	AC008608.2	
hsa-miR-3666	AC109466.1	
hsa-miR-3666	AC008429.1	

hsa-miR-3666	ZNF346-IT1	
hsa-miR-3666	AL590004.4	
hsa-miR-3666	AL137003.2	
hsa-miR-3666	E2F3-IT1	
hsa-miR-3666	HCG18	
hsa-miR-3666	AC019205.1	
hsa-miR-3666	TARID	
hsa-miR-3666	HYMAI	
hsa-miR-3666	AL022069.1	
hsa-miR-3666	Z94721.2	
hsa-miR-3666	HOXA11-AS	
hsa-miR-3666	DLX6-AS1	
hsa-miR-3666	ST7-AS1	
hsa-miR-3666	C8orf49	
hsa-miR-3666	AP006248.5	
hsa-miR-3666	AC105206.1	
hsa-miR-3666	AC107959.2	
hsa-miR-3666	RBPMS-AS1	
hsa-miR-3666	AC130304.1	
hsa-miR-3666	AC011632.1	
hsa-miR-3666	AF117829.1	
hsa-miR-3666	AC084346.2	
hsa-miR-3666	AP003469.4	
hsa-miR-3666	BAALC-AS1	
hsa-miR-3666	AC079061.1	
hsa-miR-3666	PCAT1	
hsa-miR-3666	CASC19	
hsa-miR-3666	AC107375.1	
hsa-miR-3666	AL354707.1	
hsa-miR-3666	AL158206.1	
hsa-miR-3666	AL158825.2	
hsa-miR-3666	AL162586.1	
hsa-miR-3666	SLC25A25-AS1	
hsa-miR-3666	AL359091.4	
hsa-miR-3666	LINC01503	
hsa-miR-3666	LINC00963	
hsa-miR-3666	PRKCQ-AS1	
hsa-miR-3666	AL390318.1	
hsa-miR-3666	LINC00839	
hsa-miR-3666	RP11-592B15.3	
hsa-miR-3666	NUTM2B-AS1	
hsa-miR-3666	AL359195.2	
hsa-miR-3666	AC067750.1	
hsa-miR-3666	RPARP-AS1	
hsa-miR-3666	AC069287.2	
hsa-miR-3666	AP006621.5	
hsa-miR-3666	H19	
hsa-miR-3666	KCNQ1OT1	
hsa-miR-3666	LINC00958	
hsa-miR-3666	AC068860.1	
hsa-miR-3666	LINC00294	
hsa-miR-3666	AP000437.1	
hsa-miR-3666	AP001453.1	
hsa-miR-3666	AP003068.4	
hsa-miR-3666	NEAT1	
hsa-miR-3666	AP002807.1	
hsa-miR-3666	AP000808.1	
hsa-miR-3666	AP000560.1	
hsa-miR-3666	KCTD21-AS1	
hsa-miR-3666	AP002840.2	
hsa-miR-3666	MIR100HG	
hsa-miR-3666	AP003486.1	
hsa-miR-3666	AC021054.1	
hsa-miR-3666	AC006206.2	
hsa-miR-3666	AC010186.4	
hsa-miR-3666	AC092821.3	
hsa-miR-3666	AC092828.1	
hsa-miR-3666	AC048344.4	
hsa-miR-3666	AC026356.2	
hsa-miR-3666	AC048341.2	
hsa-miR-3666	AC020656.2	
hsa-miR-3666	RMST	
hsa-miR-3666	HELLPAR	

hsa-miR-3666	AC079174.2	
hsa-miR-3666	LINC01486	
hsa-miR-3666	LINC00173	
hsa-miR-3666	AC004812.2	
hsa-miR-3666	LINC01089	
hsa-miR-3666	AC073857.1	
hsa-miR-3666	AC108704.2	
hsa-miR-3666	AC073578.3	
hsa-miR-3666	AC131009.4	
hsa-miR-3666	AC131212.3	
hsa-miR-3666	FRY-AS1	
hsa-miR-3666	AL138820.1	
hsa-miR-3666	TUSC8	
hsa-miR-3666	TPT1-AS1	
hsa-miR-3666	MIR17HG	
hsa-miR-3666	LINC01232	
hsa-miR-3666	AL355338.1	
hsa-miR-3666	RP11-397O8.7	
hsa-miR-3666	LINC00346	
hsa-miR-3666	DHRS4-AS1	
hsa-miR-3666	AL391152.1	
hsa-miR-3666	AC004817.3	
hsa-miR-3666	AC005519.1	
hsa-miR-3666	VASH1-AS1	
hsa-miR-3666	AL121768.1	
hsa-miR-3666	AL163051.2	
hsa-miR-3666	MEG8	
hsa-miR-3666	AL049840.4	
hsa-miR-3666	PWAR6	
hsa-miR-3666	SNHG14	
hsa-miR-3666	AC127502.2	
hsa-miR-3666	AC025678.3	
hsa-miR-3666	AC020658.3	
hsa-miR-3666	AC087721.1	
hsa-miR-3666	AC018362.1	
hsa-miR-3666	GABPB1-AS1	
hsa-miR-3666	AC066613.1	
hsa-miR-3666	AC025271.4	
hsa-miR-3666	AC105036.3	
hsa-miR-3666	AC019294.2	
hsa-miR-3666	AC068870.1	
hsa-miR-3666	AC068870.3	
hsa-miR-3666	AC013391.2	
hsa-miR-3666	NR2F2-AS1	
hsa-miR-3666	AC118658.2	
hsa-miR-3666	AL008727.1	
hsa-miR-3666	AL031710.2	
hsa-miR-3666	AC106820.4	
hsa-miR-3666	LINC01569	
hsa-miR-3666	AC005632.2	
hsa-miR-3666	AC092338.2	
hsa-miR-3666	AC135048.1	
hsa-miR-3666	AC135050.6	
hsa-miR-3666	AC040160.2	
hsa-miR-3666	AC004943.2	
hsa-miR-3666	AC092718.2	
hsa-miR-3666	AC092139.1	
hsa-miR-3666	AC105429.1	
hsa-miR-3666	AC015799.1	
hsa-miR-3666	AC005224.3	
hsa-miR-3666	LRRC75A-AS1	
hsa-miR-3666	LINC02076	
hsa-miR-3666	AC107983.2	
hsa-miR-3666	CCDC144NL-AS1	
hsa-miR-3666	AC005288.1	
hsa-miR-3666	AC008105.3	
hsa-miR-3666	AC091133.1	
hsa-miR-3666	AC005921.3	
hsa-miR-3666	AC005839.1	
hsa-miR-3666	AC015813.6	
hsa-miR-3666	AC120024.4	
hsa-miR-3666	BAIAP2-AS1	
hsa-miR-3666	AC124283.3	

hsa-miR-3666	LINC00667	
hsa-miR-3666	LINC01887	
hsa-miR-3666	AP001029.2	
hsa-miR-3666	AC007996.1	
hsa-miR-3666	AC100847.1	
hsa-miR-3666	AC105105.2	
hsa-miR-3666	AC105105.1	
hsa-miR-3666	AC023301.1	
hsa-miR-3666	AC009716.2	
hsa-miR-3666	AC114341.1	
hsa-miR-3666	AC006538.1	
hsa-miR-3666	AC011461.1	
hsa-miR-3666	ILF3-AS1	
hsa-miR-3666	AC008543.3	
hsa-miR-3666	BISPR	
hsa-miR-3666	LINC00663	
hsa-miR-3666	AC011503.2	
hsa-miR-3666	LINC00662	
hsa-miR-3666	AC007786.3	
hsa-miR-3666	AC021092.1	
hsa-miR-3666	AC010325.2	
hsa-miR-3666	AC012313.3	
hsa-miR-3666	AL117335.1	
hsa-miR-3666	LINC00261	
hsa-miR-3666	AL121827.2	
hsa-miR-3666	UCKL1-AS1	
hsa-miR-3666	AF127936.2	
hsa-miR-3666	BACH1-IT2	
hsa-miR-3666	LINC02575	
hsa-miR-3666	MCM3AP-AS1	
hsa-miR-3666	AP001469.3	
hsa-miR-3666	AC016027.2	
hsa-miR-3666	MIAT	
hsa-miR-3666	AC005005.4	
hsa-miR-3666	LINC01521	
hsa-miR-3666	Z95331.1	
hsa-miR-3666	AL021392.1	
hsa-miR-3666	FP325330.3	
hsa-miR-3666	PINCR	
hsa-miR-3666	LINC01278	
hsa-miR-3666	XIST	
hsa-miR-3666	AL359740.1	
hsa-miR-3666	Z83843.1	
hsa-miR-3666	AL035425.4	
hsa-miR-3666	AL683813.1	
hsa-miR-3666	AC245140.1	
hsa-miR-422a	LINC01128	
hsa-miR-422a	AL645608.3	
hsa-miR-422a	AL645608.1	
hsa-miR-422a	AL391244.1	
hsa-miR-422a	AL590438.1	
hsa-miR-422a	MIR34AHG	
hsa-miR-422a	AC254633.1	
hsa-miR-422a	AL451042.2	
hsa-miR-422a	LINC01772	
hsa-miR-422a	PINK1-AS	
hsa-miR-422a	AL049795.2	
hsa-miR-422a	AL662907.2	
hsa-miR-422a	NFYC-AS1	
hsa-miR-422a	AL512353.1	
hsa-miR-422a	SZT2-AS1	
hsa-miR-422a	KDM4A-AS1	
hsa-miR-422a	LINC01358	
hsa-miR-422a	LINC01748	
hsa-miR-422a	RP4-565E6.1	
hsa-miR-422a	XXyac-YX155B6.6	
hsa-miR-422a	AC234582.1	
hsa-miR-422a	RUSC1-AS1	
hsa-miR-422a	KIRREL1-IT1	
hsa-miR-422a	LINC01133	
hsa-miR-422a	AL592435.1	
hsa-miR-422a	DNM3OS	
hsa-miR-422a	MIR29B2CHG	

hsa-miR-422a	AKT3-IT1	
hsa-miR-422a	C2orf48	
hsa-miR-422a	RN7SL832P	
hsa-miR-422a	LINC01126	
hsa-miR-422a	AC093110.1	
hsa-miR-422a	LINC02245	
hsa-miR-422a	AC005041.3	
hsa-miR-422a	AC012360.3	
hsa-miR-422a	MIR4435-2HG	
hsa-miR-422a	PAX8-AS1	
hsa-miR-422a	AC110769.1	
hsa-miR-422a	AC073050.1	
hsa-miR-422a	AC016723.1	
hsa-miR-422a	AC009948.5	
hsa-miR-422a	AC016708.1	
hsa-miR-422a	THUMPD3-AS1	
hsa-miR-422a	AC090948.2	
hsa-miR-422a	ITGA9-AS1	
hsa-miR-422a	AC104447.1	
hsa-miR-422a	RASSF1-AS1	
hsa-miR-422a	ADAMTS9-AS2	
hsa-miR-422a	DUBR	
hsa-miR-422a	LINC01205	
hsa-miR-422a	AC112503.2	
hsa-miR-422a	AC022336.3	
hsa-miR-422a	MBNL1-AS1	
hsa-miR-422a	AC073288.1	
hsa-miR-422a	YEATS2-AS1	
hsa-miR-422a	MELTF-AS1	
hsa-miR-422a	AC139887.2	
hsa-miR-422a	CTBP1-AS2	
hsa-miR-422a	AC097382.2	
hsa-miR-422a	AC007370.2	
hsa-miR-422a	AC107068.1	
hsa-miR-422a	UBA6-AS1	
hsa-miR-422a	AC105285.1	
hsa-miR-422a	AC010442.1	
hsa-miR-422a	AC010491.1	
hsa-miR-422a	BASP1-AS1	
hsa-miR-422a	STARD4-AS1	
hsa-miR-422a	AC116366.1	
hsa-miR-422a	TRIM52-AS1	
hsa-miR-422a	AL355336.1	
hsa-miR-422a	Z97832.2	
hsa-miR-422a	AL035587.1	
hsa-miR-422a	LINC00472	
hsa-miR-422a	AC019205.1	
hsa-miR-422a	AL596202.1	
hsa-miR-422a	AL354892.3	
hsa-miR-422a	AC147651.1	
hsa-miR-422a	PSMG3-AS1	
hsa-miR-422a	AC004691.1	
hsa-miR-422a	POLR2J4	
hsa-miR-422a	EGFR-AS1	
hsa-miR-422a	AC118758.3	
hsa-miR-422a	AC073188.5	
hsa-miR-422a	LINC00174	
hsa-miR-422a	AC027644.3	
hsa-miR-422a	DTX2P1-UPK3BP1-PMS2P11	
hsa-miR-422a	MAGI2-AS3	
hsa-miR-422a	AC002076.1	
hsa-miR-422a	STAG3L5P-PVRIG2P-PILRB	
hsa-miR-422a	AC092849.1	
hsa-miR-422a	AC000123.3	
hsa-miR-422a	AC016831.1	
hsa-miR-422a	AC008264.2	
hsa-miR-422a	JHDM1D-AS1	
hsa-miR-422a	PAXIP1-AS1	
hsa-miR-422a	AC107959.2	
hsa-miR-422a	AF106564.1	
hsa-miR-422a	AC124067.4	
hsa-miR-422a	MSC-AS1	
hsa-miR-422a	AF117829.1	

hsa-miR-422a	AZIN1-AS1	
hsa-miR-422a	PRNCR1	
hsa-miR-422a	AC105235.1	
hsa-miR-422a	EBLN3P	
hsa-miR-422a	AL158071.4	
hsa-miR-422a	AL589843.1	
hsa-miR-422a	AL512590.3	
hsa-miR-422a	AL358074.1	
hsa-miR-422a	LINC00963	
hsa-miR-422a	PPP1R26-AS1	
hsa-miR-422a	AL355987.4	
hsa-miR-422a	ARRDC1-AS1	
hsa-miR-422a	AL359878.1	
hsa-miR-422a	AL139125.2	
hsa-miR-422a	AL157392.3	
hsa-miR-422a	RP11-292F22.7	
hsa-miR-422a	AC069547.1	
hsa-miR-422a	AC016822.1	
hsa-miR-422a	AL132656.3	
hsa-miR-422a	AC067750.1	
hsa-miR-422a	ACTA2-AS1	
hsa-miR-422a	OLMALINC	
hsa-miR-422a	AC025947.1	
hsa-miR-422a	AP006621.4	
hsa-miR-422a	KCNQ1OT1	
hsa-miR-422a	LINC02547	
hsa-miR-422a	LINC00958	
hsa-miR-422a	AC090833.1	
hsa-miR-422a	AP003733.4	
hsa-miR-422a	NEAT1	
hsa-miR-422a	MALAT1	
hsa-miR-422a	AP003419.2	
hsa-miR-422a	AP000590.1	
hsa-miR-422a	AP001972.5	
hsa-miR-422a	AP002840.2	
hsa-miR-422a	BACE1-AS	
hsa-miR-422a	AP003392.5	
hsa-miR-422a	MIR100HG	
hsa-miR-422a	LINC00987	
hsa-miR-422a	RASSF8-AS1	
hsa-miR-422a	AC008124.1	
hsa-miR-422a	AC025031.4	
hsa-miR-422a	AC023509.1	
hsa-miR-422a	AGAP2-AS1	
hsa-miR-422a	AC026124.1	
hsa-miR-422a	AC025423.4	
hsa-miR-422a	AC010203.2	
hsa-miR-422a	HELLPAR	
hsa-miR-422a	NRAV	
hsa-miR-422a	AC122688.3	
hsa-miR-422a	AC108704.2	
hsa-miR-422a	AC131212.3	
hsa-miR-422a	LINC02344	
hsa-miR-422a	AL161421.1	
hsa-miR-422a	AL137782.1	
hsa-miR-422a	LINC00346	
hsa-miR-422a	ANKRD10-IT1	
hsa-miR-422a	LINC00641	
hsa-miR-422a	LINC02313	
hsa-miR-422a	AL139099.4	
hsa-miR-422a	AL139099.1	
hsa-miR-422a	AL627171.1	
hsa-miR-422a	PSMA3-AS1	
hsa-miR-422a	AL133370.1	
hsa-miR-422a	SNHG10	
hsa-miR-422a	AL135838.1	
hsa-miR-422a	SNHG14	
hsa-miR-422a	AC124312.4	
hsa-miR-422a	PWAR5	
hsa-miR-422a	AC025678.3	
hsa-miR-422a	AC087516.2	
hsa-miR-422a	AC116158.1	
hsa-miR-422a	AC013652.2	

hsa-miR-422a	OIP5-AS1	
hsa-miR-422a	GABPB1-IT1	
hsa-miR-422a	GABPB1-AS1	
hsa-miR-422a	LINC00926	
hsa-miR-422a	TPM1-AS	
hsa-miR-422a	AC007950.2	
hsa-miR-422a	AC110048.2	
hsa-miR-422a	AC090826.1	
hsa-miR-422a	UBL7-AS1	
hsa-miR-422a	AC023024.1	
hsa-miR-422a	Z92544.2	
hsa-miR-422a	AC009065.4	
hsa-miR-422a	AC093525.8	
hsa-miR-422a	AC093525.7	
hsa-miR-422a	AC092117.2	
hsa-miR-422a	SRRM2-AS1	
hsa-miR-422a	AC131649.2	
hsa-miR-422a	IL21R-AS1	
hsa-miR-422a	AC138904.1	
hsa-miR-422a	MIR762HG	
hsa-miR-422a	FBXL19-AS1	
hsa-miR-422a	AC135048.1	
hsa-miR-422a	AC009102.2	
hsa-miR-422a	AC010542.4	
hsa-miR-422a	AC027682.6	
hsa-miR-422a	AC116667.1	
hsa-miR-422a	AC009078.3	
hsa-miR-422a	AC092139.3	
hsa-miR-422a	AC009063.2	
hsa-miR-422a	ATP2C2-AS1	
hsa-miR-422a	FENDRR	
hsa-miR-422a	AC027763.2	
hsa-miR-422a	MYHAS	
hsa-miR-422a	SPAG5-AS1	
hsa-miR-422a	AC068669.1	
hsa-miR-422a	AC067852.3	
hsa-miR-422a	AC067852.2	
hsa-miR-422a	AC060780.1	
hsa-miR-422a	ASB16-AS1	
hsa-miR-422a	AC002558.3	
hsa-miR-422a	AC005920.1	
hsa-miR-422a	AC007485.2	
hsa-miR-422a	CTC-462L7.1	
hsa-miR-422a	AC015813.1	
hsa-miR-422a	AC079331.1	
hsa-miR-422a	AC005332.6	
hsa-miR-422a	TEN1-CDK3	
hsa-miR-422a	SNHG16	
hsa-miR-422a	AC111170.2	
hsa-miR-422a	BAIAP2-AS1	
hsa-miR-422a	AC110285.1	
hsa-miR-422a	AC139530.1	
hsa-miR-422a	AC132872.4	
hsa-miR-422a	AC132872.1	
hsa-miR-422a	AC124283.3	
hsa-miR-422a	TYMSOS	
hsa-miR-422a	AP005057.1	
hsa-miR-422a	AP005131.6	
hsa-miR-422a	AC011815.1	
hsa-miR-422a	AC007998.3	
hsa-miR-422a	AC105105.2	
hsa-miR-422a	AC036176.1	
hsa-miR-422a	AC093330.2	
hsa-miR-422a	AC007292.2	
hsa-miR-422a	AC007292.1	
hsa-miR-422a	AC008760.1	
hsa-miR-422a	AC020951.1	
hsa-miR-422a	BISPR	
hsa-miR-422a	AC092279.1	
hsa-miR-422a	LINC00662	
hsa-miR-422a	AC006504.5	
hsa-miR-422a	AC079466.1	
hsa-miR-422a	TMEM147-AS1	

hsa-miR-422a	AC016590.3	
hsa-miR-422a	AC118344.1	
hsa-miR-422a	AC006486.2	
hsa-miR-422a	AC007191.1	
hsa-miR-422a	AC007228.2	
hsa-miR-422a	AC005261.3	
hsa-miR-422a	AC005261.1	
hsa-miR-422a	AC012313.5	
hsa-miR-422a	MZF1-AS1	
hsa-miR-422a	SDCBP2-AS1	
hsa-miR-422a	AL109976.1	
hsa-miR-422a	AL121906.2	
hsa-miR-422a	AL121753.2	
hsa-miR-422a	NORAD	
hsa-miR-422a	ZFAS1	
hsa-miR-422a	APCDD1L-AS1	
hsa-miR-422a	AL118506.1	
hsa-miR-422a	AF127577.4	
hsa-miR-422a	LINC02246	
hsa-miR-422a	LINC00205	
hsa-miR-422a	GUSBP11	
hsa-miR-422a	AP000347.2	
hsa-miR-422a	MIAT	
hsa-miR-422a	LINC01422	
hsa-miR-422a	AL022311.1	
hsa-miR-422a	Z95331.1	
hsa-miR-422a	MIRLET7BHG	
hsa-miR-422a	AL117329.1	
hsa-miR-422a	Z97192.3	
hsa-miR-422a	C22orf34	
hsa-miR-422a	BX890604.1	
hsa-miR-422a	FAM239B	
hsa-miR-422a	AC073529.1	
hsa-miR-422a	AC004656.1	
hsa-miR-422a	JPX	
hsa-miR-422a	FTX	
hsa-miR-451a	AL033528.2	
hsa-miR-451a	SNHG12	
hsa-miR-451a	TDRKH-AS1	
hsa-miR-451a	AL355388.2	
hsa-miR-451a	AC007743.1	
hsa-miR-451a	AC005041.3	
hsa-miR-451a	AC098820.1	
hsa-miR-451a	AC005538.2	
hsa-miR-451a	AC108673.3	
hsa-miR-451a	ZSCAN16-AS1	
hsa-miR-451a	PSMG3-AS1	
hsa-miR-451a	AC017116.1	
hsa-miR-451a	SNHG15	
hsa-miR-451a	POT1-AS1	
hsa-miR-451a	AC007938.2	
hsa-miR-451a	AP000753.2	
hsa-miR-451a	AC006206.1	
hsa-miR-451a	SOX21-AS1	
hsa-miR-451a	SLC25A21-AS1	
hsa-miR-451a	AC022167.2	
hsa-miR-451a	LINC01290	
hsa-miR-451a	AC135050.7	
hsa-miR-451a	AC092134.2	
hsa-miR-451a	AC092127.1	
hsa-miR-451a	AC137932.1	
hsa-miR-451a	ARHGAP27P1-BPTFP1-KPNA2P3	
hsa-miR-451a	DLGAP1-AS2	
hsa-miR-451a	AC092296.1	
hsa-miR-451a	NORAD	
hsa-miR-451a	SNHG17	
hsa-miR-451a	AC073529.1	
hsa-miR-451a	LINC01278	
hsa-miR-551a	LINC01128	
hsa-miR-551a	AL031283.1	
hsa-miR-551a	AL359258.1	
hsa-miR-551a	AL390038.1	
hsa-miR-551a	RBM15-AS1	

hsa-miR-551a	FALEC	
hsa-miR-551a	AL365436.2	
hsa-miR-551a	AL365181.3	
hsa-miR-551a	AL390728.5	
hsa-miR-551a	AC009948.1	
hsa-miR-551a	AC011298.1	
hsa-miR-551a	VIPR1-AS1	
hsa-miR-551a	AC022336.2	
hsa-miR-551a	AC105285.1	
hsa-miR-551a	SLC9A3-AS1	
hsa-miR-551a	LINC01023	
hsa-miR-551a	AC008443.4	
hsa-miR-551a	SYNJ2-IT1	
hsa-miR-551a	AC147651.1	
hsa-miR-551a	DLX6-AS1	
hsa-miR-551a	STAG3L5P-PVRIG2P-PILRB	
hsa-miR-551a	AC069281.2	
hsa-miR-551a	AC005229.4	
hsa-miR-551a	LINC00599	
hsa-miR-551a	EXTL3-AS1	
hsa-miR-551a	LINC01301	
hsa-miR-551a	PVT1	
hsa-miR-551a	LINC01242	
hsa-miR-551a	NALT1	
hsa-miR-551a	AL359878.1	
hsa-miR-551a	NUTM2B-AS1	
hsa-miR-551a	AP001972.5	
hsa-miR-551a	COLCA1	
hsa-miR-551a	AP004609.3	
hsa-miR-551a	AP003392.3	
hsa-miR-551a	U47924.2	
hsa-miR-551a	AC025569.1	
hsa-miR-551a	AC124947.1	
hsa-miR-551a	HELLPAR	
hsa-miR-551a	AC007541.1	
hsa-miR-551a	AC026368.1	
hsa-miR-551a	AL162377.1	
hsa-miR-551a	AL096870.2	
hsa-miR-551a	AL162311.3	
hsa-miR-551a	USP3-AS1	
hsa-miR-551a	AC009041.3	
hsa-miR-551a	AC009090.4	
hsa-miR-551a	AC126696.1	
hsa-miR-551a	AC005899.4	
hsa-miR-551a	AC090627.1	
hsa-miR-551a	HOXB-AS3	
hsa-miR-551a	LINC01915	
hsa-miR-551a	AC005944.1	
hsa-miR-551a	AC008894.3	
hsa-miR-551a	ZNF582-AS1	
hsa-miR-551a	PLCG1-AS1	
hsa-miR-551a	LINC01666	
hsa-miR-551a	LINC00649	
hsa-miR-551a	LINC01547	
hsa-miR-551a	MIAT	
hsa-miR-551a	Z93930.3	
hsa-miR-551a	AL079301.1	
hsa-miR-551a	Z93022.1	
hsa-miR-650	AL645608.8	
hsa-miR-650	AL391244.1	
hsa-miR-650	AL031282.2	
hsa-miR-650	AL953897.1	
hsa-miR-650	LINC01772	
hsa-miR-650	PINK1-AS	
hsa-miR-650	SNHG3	
hsa-miR-650	SNHG12	
hsa-miR-650	AL049795.2	
hsa-miR-650	AL139260.2	
hsa-miR-650	SLFNL1-AS1	
hsa-miR-650	AL139220.2	
hsa-miR-650	LINC01144	
hsa-miR-650	AL353898.3	
hsa-miR-650	LINC01748	

hsa-miR-650	CCDC18-AS1	
hsa-miR-650	AL109613.1	
hsa-miR-650	AL160006.1	
hsa-miR-650	SLC16A1-AS1	
hsa-miR-650	AL359915.2	
hsa-miR-650	RP4-565E6.1	
hsa-miR-650	AC239809.3	
hsa-miR-650	XXyac-YX155B6.6	
hsa-miR-650	TDRKH-AS1	
hsa-miR-650	AC234582.1	
hsa-miR-650	ASH1L-AS1	
hsa-miR-650	AL365181.2	
hsa-miR-650	AL590666.3	
hsa-miR-650	AL121987.2	
hsa-miR-650	GAS5	
hsa-miR-650	C1orf220	
hsa-miR-650	MIR181A1HG	
hsa-miR-650	AL691482.3	
hsa-miR-650	C1orf147	
hsa-miR-650	MIR29B2CHG	
hsa-miR-650	FLVCR1-AS1	
hsa-miR-650	AL670729.1	
hsa-miR-650	LINC01736	
hsa-miR-650	AL512328.1	
hsa-miR-650	AL359921.2	
hsa-miR-650	LINC01250	
hsa-miR-650	EIPR1-IT1	
hsa-miR-650	ID2-AS1	
hsa-miR-650	AC082651.1	
hsa-miR-650	AC012073.1	
hsa-miR-650	AC074117.1	
hsa-miR-650	AL121658.1	
hsa-miR-650	AC012358.1	
hsa-miR-650	PCBP1-AS1	
hsa-miR-650	ALMS1-IT1	
hsa-miR-650	AC092653.2	
hsa-miR-650	ST3GAL5-AS1	
hsa-miR-650	LINC00342	
hsa-miR-650	LINC01158	
hsa-miR-650	AC068491.4	
hsa-miR-650	PAX8-AS1	
hsa-miR-650	AC009948.1	
hsa-miR-650	AC009948.5	
hsa-miR-650	TTN-AS1	
hsa-miR-650	LINC01473	
hsa-miR-650	AC007383.4	
hsa-miR-650	AC012510.1	
hsa-miR-650	AC097461.1	
hsa-miR-650	AC073476.3	
hsa-miR-650	AC105760.2	
hsa-miR-650	AC104667.2	
hsa-miR-650	AC069277.1	
hsa-miR-650	THUMPD3-AS1	
hsa-miR-650	AC018809.2	
hsa-miR-650	ACVR2B-AS1	
hsa-miR-650	AC099668.1	
hsa-miR-650	U73166.1	
hsa-miR-650	AC012467.2	
hsa-miR-650	PRICKLE2-AS1	
hsa-miR-650	FOXP1-IT1	
hsa-miR-650	ZBTB11-AS1	
hsa-miR-650	DUBR	
hsa-miR-650	LINC02035	
hsa-miR-650	GATA2-AS1	
hsa-miR-650	NCK1-AS1	
hsa-miR-650	AC107021.2	
hsa-miR-650	AC080013.1	
hsa-miR-650	AC022498.2	
hsa-miR-650	LINC00885	
hsa-miR-650	AC092535.1	
hsa-miR-650	CTBP1-AS2	
hsa-miR-650	AC016773.1	
hsa-miR-650	LINC02447	

hsa-miR-650	SLIT2-IT1	
hsa-miR-650	AC107068.1	
hsa-miR-650	EPHA5-AS1	
hsa-miR-650	AC110760.2	
hsa-miR-650	AC021127.1	
hsa-miR-650	THAP9-AS1	
hsa-miR-650	AC093895.1	
hsa-miR-650	LEF1-AS1	
hsa-miR-650	AC106895.2	
hsa-miR-650	LINC02365	
hsa-miR-650	SLC9A3-AS1	
hsa-miR-650	LINC01033	
hsa-miR-650	AC010501.1	
hsa-miR-650	LUCAT1	
hsa-miR-650	AC106786.1	
hsa-miR-650	AC106791.1	
hsa-miR-650	SNHG4	
hsa-miR-650	AC116353.4	
hsa-miR-650	AC005592.1	
hsa-miR-650	PRR7-AS1	
hsa-miR-650	AC145098.2	
hsa-miR-650	AC139795.2	
hsa-miR-650	AC138035.1	
hsa-miR-650	SERPINB9P1	
hsa-miR-650	AL031963.3	
hsa-miR-650	AL138881.1	
hsa-miR-650	AL391422.3	
hsa-miR-650	AL590004.3	
hsa-miR-650	MIR5689HG	
hsa-miR-650	AL136162.1	
hsa-miR-650	AL009031.1	
hsa-miR-650	AL031775.1	
hsa-miR-650	ZSCAN16-AS1	
hsa-miR-650	HCP5	
hsa-miR-650	AL354740.1	
hsa-miR-650	AL121574.1	
hsa-miR-650	AL592158.1	
hsa-miR-650	TRAM2-AS1	
hsa-miR-650	LINC00472	
hsa-miR-650	MANEA-AS1	
hsa-miR-650	AL023581.2	
hsa-miR-650	AL358972.1	
hsa-miR-650	LINC00473	
hsa-miR-650	PSMG3-AS1	
hsa-miR-650	AC092171.5	
hsa-miR-650	AC007128.2	
hsa-miR-650	HOXA11-AS	
hsa-miR-650	AC005154.1	
hsa-miR-650	LINC00265	
hsa-miR-650	AC118758.3	
hsa-miR-650	LINC00174	
hsa-miR-650	AC073335.2	
hsa-miR-650	AC003991.1	
hsa-miR-650	AC002076.1	
hsa-miR-650	DLX6-AS1	
hsa-miR-650	STAG3L5P-PVRIG2P-PILRB	
hsa-miR-650	KMT2E-AS1	
hsa-miR-650	SLC26A4-AS1	
hsa-miR-650	ST7-OT4	
hsa-miR-650	AC016831.1	
hsa-miR-650	LINC-PINT	
hsa-miR-650	AC016831.7	
hsa-miR-650	LINC01003	
hsa-miR-650	LINC00689	
hsa-miR-650	AF131215.5	
hsa-miR-650	AF131215.4	
hsa-miR-650	AC087821.1	
hsa-miR-650	AC037459.3	
hsa-miR-650	AC064807.1	
hsa-miR-650	AC009812.3	
hsa-miR-650	AF117829.1	
hsa-miR-650	OTUD6B-AS1	
hsa-miR-650	AP003476.1	

hsa-miR-650	HAS2-AS1	
hsa-miR-650	AC107375.1	
hsa-miR-650	AC084125.2	
hsa-miR-650	VLDLR-AS1	
hsa-miR-650	AL359095.1	
hsa-miR-650	PTPRD-AS1	
hsa-miR-650	AL353795.2	
hsa-miR-650	AL513165.1	
hsa-miR-650	SMC5-AS1	
hsa-miR-650	AL353726.2	
hsa-miR-650	AL590705.5	
hsa-miR-650	AL512590.3	
hsa-miR-650	AL138756.1	
hsa-miR-650	AL358074.1	
hsa-miR-650	AL162586.1	
hsa-miR-650	SLC25A25-AS1	
hsa-miR-650	LINC00963	
hsa-miR-650	AL138781.1	
hsa-miR-650	C9orf163	
hsa-miR-650	CCDC183-AS1	
hsa-miR-650	STPG3-AS1	
hsa-miR-650	BX255925.1	
hsa-miR-650	AL139125.2	
hsa-miR-650	AL157392.3	
hsa-miR-650	ZEB1-AS1	
hsa-miR-650	AL117336.3	
hsa-miR-650	AL353801.3	
hsa-miR-650	AC022532.1	
hsa-miR-650	AL731563.3	
hsa-miR-650	PPP3CB-AS1	
hsa-miR-650	AC010997.4	
hsa-miR-650	NUTM2B-AS1	
hsa-miR-650	ADIRF-AS1	
hsa-miR-650	NUTM2A-AS1	
hsa-miR-650	AL391684.1	
hsa-miR-650	LBX1-AS1	
hsa-miR-650	AL133355.1	
hsa-miR-650	PDCD4-AS1	
hsa-miR-650	AL158212.3	
hsa-miR-650	AL157832.1	
hsa-miR-650	AL360181.2	
hsa-miR-650	AP006621.5	
hsa-miR-650	AC124057.1	
hsa-miR-650	KCNQ1OT1	
hsa-miR-650	AC091564.7	
hsa-miR-650	TMEM9B-AS1	
hsa-miR-650	SBF2-AS1	
hsa-miR-650	AC124798.1	
hsa-miR-650	AC068205.2	
hsa-miR-650	AP001350.2	
hsa-miR-650	SNHG1	
hsa-miR-650	NEAT1	
hsa-miR-650	MALAT1	
hsa-miR-650	AP002490.1	
hsa-miR-650	AP002761.4	
hsa-miR-650	AP003717.1	
hsa-miR-650	MIR4300HG	
hsa-miR-650	AP000446.1	
hsa-miR-650	AP000766.1	
hsa-miR-650	COLCA1	
hsa-miR-650	BACE1-AS	
hsa-miR-650	MIR100HG	
hsa-miR-650	SENCR	
hsa-miR-650	AP003486.1	
hsa-miR-650	AC004803.1	
hsa-miR-650	AC125807.2	
hsa-miR-650	AC022075.1	
hsa-miR-650	AC023790.2	
hsa-miR-650	AC010168.2	
hsa-miR-650	AC026356.2	
hsa-miR-650	AC008014.1	
hsa-miR-650	LINC02396	
hsa-miR-650	KRT73-AS1	

hsa-miR-650	AC012531.2	
hsa-miR-650	LINC02381	
hsa-miR-650	AC023794.7	
hsa-miR-650	AC009779.2	
hsa-miR-650	AGAP2-AS1	
hsa-miR-650	AC020656.2	
hsa-miR-650	HELLPAR	
hsa-miR-650	AC007622.2	
hsa-miR-650	NRAV	
hsa-miR-650	LINC01089	
hsa-miR-650	AC026362.1	
hsa-miR-650	AC068768.1	
hsa-miR-650	AC055713.1	
hsa-miR-650	AL445985.1	
hsa-miR-650	LINC00571	
hsa-miR-650	AL162713.1	
hsa-miR-650	TUSC8	
hsa-miR-650	LINC00330	
hsa-miR-650	LINC00355	
hsa-miR-650	AL445223.1	
hsa-miR-650	AL161431.1	
hsa-miR-650	LINC00346	
hsa-miR-650	AL355075.4	
hsa-miR-650	ZFHX2-AS1	
hsa-miR-650	AL079305.1	
hsa-miR-650	AL079303.1	
hsa-miR-650	AL627171.2	
hsa-miR-650	AL358332.1	
hsa-miR-650	LINC02331	
hsa-miR-650	PSMA3-AS1	
hsa-miR-650	AL161756.1	
hsa-miR-650	VASH1-AS1	
hsa-miR-650	AL049775.1	
hsa-miR-650	LINC01146	
hsa-miR-650	AL162171.3	
hsa-miR-650	AL137230.2	
hsa-miR-650	DICER1-AS1	
hsa-miR-650	MEG3	
hsa-miR-650	MEG8	
hsa-miR-650	LINC02323	
hsa-miR-650	AL049840.4	
hsa-miR-650	AL928654.4	
hsa-miR-650	AC124312.3	
hsa-miR-650	SNHG14	
hsa-miR-650	AC068448.1	
hsa-miR-650	AC087516.2	
hsa-miR-650	AC023908.2	
hsa-miR-650	AC022405.1	
hsa-miR-650	AC025271.4	
hsa-miR-650	AC103691.1	
hsa-miR-650	AC110048.2	
hsa-miR-650	DRAIC	
hsa-miR-650	AC015871.4	
hsa-miR-650	MIR9-3HG	
hsa-miR-650	ZNF710-AS1	
hsa-miR-650	AC091078.1	
hsa-miR-650	AC103746.1	
hsa-miR-650	AC087477.2	
hsa-miR-650	LINC00923	
hsa-miR-650	AC022819.1	
hsa-miR-650	PCSK6-AS1	
hsa-miR-650	LINC02348	
hsa-miR-650	Z92544.2	
hsa-miR-650	AL008727.1	
hsa-miR-650	AC009041.4	
hsa-miR-650	AL133297.2	
hsa-miR-650	AC106820.5	
hsa-miR-650	AC106820.4	
hsa-miR-650	AC093525.8	
hsa-miR-650	AC093525.7	
hsa-miR-650	AC141586.5	
hsa-miR-650	SRRM2-AS1	
hsa-miR-650	AC092117.1	

hsa-miR-650	AC004233.3	
hsa-miR-650	MMP25-AS1	
hsa-miR-650	AC012676.5	
hsa-miR-650	AC022167.2	
hsa-miR-650	AC133065.3	
hsa-miR-650	AC009121.2	
hsa-miR-650	AC009152.1	
hsa-miR-650	LINC02175	
hsa-miR-650	AC145285.2	
hsa-miR-650	AC109460.3	
hsa-miR-650	SLX1B-SULT1A4	
hsa-miR-650	AC009133.2	
hsa-miR-650	AC120114.4	
hsa-miR-650	SLX1A-SULT1A3	
hsa-miR-650	AC093249.6	
hsa-miR-650	AC106886.2	
hsa-miR-650	MIR762HG	
hsa-miR-650	FBXL19-AS1	
hsa-miR-650	AC135048.1	
hsa-miR-650	AC007599.2	
hsa-miR-650	AC007608.4	
hsa-miR-650	AC020978.1	
hsa-miR-650	AC004943.2	
hsa-miR-650	AC009078.3	
hsa-miR-650	AC099506.1	
hsa-miR-650	AC092127.1	
hsa-miR-650	AC010536.1	
hsa-miR-650	SNAI3-AS1	
hsa-miR-650	AC015799.1	
hsa-miR-650	AC087500.1	
hsa-miR-650	MIR497HG	
hsa-miR-650	TMEM220-AS1	
hsa-miR-650	AC007952.4	
hsa-miR-650	AC007952.5	
hsa-miR-650	CCDC144NL-AS1	
hsa-miR-650	AC087294.1	
hsa-miR-650	AC233702.9	
hsa-miR-650	AC104564.5	
hsa-miR-650	AC060766.4	
hsa-miR-650	AC080112.1	
hsa-miR-650	AC018629.1	
hsa-miR-650	AC060780.1	
hsa-miR-650	ASB16-AS1	
hsa-miR-650	AC003102.1	
hsa-miR-650	RUNDC3A-AS1	
hsa-miR-650	AC138150.2	
hsa-miR-650	AC068152.1	
hsa-miR-650	AC103702.2	
hsa-miR-650	AC027801.1	
hsa-miR-650	AC005839.1	
hsa-miR-650	AC015813.6	
hsa-miR-650	TBC1D3P1-DHX40P1	
hsa-miR-650	AC018628.1	
hsa-miR-650	AC005828.4	
hsa-miR-650	ARHGAP27P1-BPTFP1-KPNA2P3	
hsa-miR-650	AC005332.7	
hsa-miR-650	AC005332.6	
hsa-miR-650	AC005332.1	
hsa-miR-650	LINC01482	
hsa-miR-650	AC097641.2	
hsa-miR-650	AC064805.1	
hsa-miR-650	SNHG16	
hsa-miR-650	LINC02080	
hsa-miR-650	SNHG20	
hsa-miR-650	AC111170.3	
hsa-miR-650	DNAH17-AS1	
hsa-miR-650	AC087741.1	
hsa-miR-650	AC027601.1	
hsa-miR-650	AC110285.2	
hsa-miR-650	AC145207.5	
hsa-miR-650	MAFG-AS1	
hsa-miR-650	AC124283.5	
hsa-miR-650	AC144831.1	

hsa-miR-650	AP001120.1	
hsa-miR-650	C18orf15	
hsa-miR-650	AC090206.1	
hsa-miR-650	AC093462.1	
hsa-miR-650	LINC01544	
hsa-miR-650	AC009704.2	
hsa-miR-650	AC093330.1	
hsa-miR-650	AC012615.1	
hsa-miR-650	AC119403.1	
hsa-miR-650	AC011498.2	
hsa-miR-650	AC011444.2	
hsa-miR-650	AC011472.5	
hsa-miR-650	AC008543.1	
hsa-miR-650	AC020917.4	
hsa-miR-650	AC138430.1	
hsa-miR-650	AC010615.2	
hsa-miR-650	LINC01224	
hsa-miR-650	LINC00662	
hsa-miR-650	AC005394.2	
hsa-miR-650	AC008555.8	
hsa-miR-650	LINC00665	
hsa-miR-650	AC016590.1	
hsa-miR-650	AC016590.2	
hsa-miR-650	AC022144.1	
hsa-miR-650	AC008982.2	
hsa-miR-650	AC118344.1	
hsa-miR-650	AC008537.3	
hsa-miR-650	AC243964.3	
hsa-miR-650	AC243964.2	
hsa-miR-650	GEMIN7-AS1	
hsa-miR-650	AC007191.1	
hsa-miR-650	AC074212.1	
hsa-miR-650	AC006262.1	
hsa-miR-650	AC010331.1	
hsa-miR-650	AC010327.3	
hsa-miR-650	CTD-2105E13.13	
hsa-miR-650	ZNF667-AS1	
hsa-miR-650	A1BG-AS1	
hsa-miR-650	AC012313.1	
hsa-miR-650	AC012313.8	
hsa-miR-650	AL121760.1	
hsa-miR-650	AL049712.1	
hsa-miR-650	AL353194.1	
hsa-miR-650	LINC00654	
hsa-miR-650	AL049649.1	
hsa-miR-650	MIR663AHG	
hsa-miR-650	RALY-AS1	
hsa-miR-650	AL136172.1	
hsa-miR-650	OSER1-AS1	
hsa-miR-650	AL117382.2	
hsa-miR-650	AL031666.1	
hsa-miR-650	LINC01273	
hsa-miR-650	LINC01271	
hsa-miR-650	LINC01524	
hsa-miR-650	GNAS-AS1	
hsa-miR-650	AL121832.3	
hsa-miR-650	AL117379.1	
hsa-miR-650	MIR155HG	
hsa-miR-650	AP000688.1	
hsa-miR-650	DSCAM-AS1	
hsa-miR-650	AP001057.1	
hsa-miR-650	AP001062.1	
hsa-miR-650	LINC01694	
hsa-miR-650	AL592528.1	
hsa-miR-650	CECR3	
hsa-miR-650	AC004019.18	
hsa-miR-650	AP000553.2	
hsa-miR-650	GUSBP11	
hsa-miR-650	AP000347.2	
hsa-miR-650	AP000350.6	
hsa-miR-650	ADORA2A-AS1	
hsa-miR-650	LINC01422	
hsa-miR-650	AC004264.2	

hsa-miR-650	TUG1	
hsa-miR-650	LINC01521	
hsa-miR-650	LARGE-IT1	
hsa-miR-650	Z82217.1	
hsa-miR-650	Z82188.2	
hsa-miR-650	AL022311.1	
hsa-miR-650	AL022322.1	
hsa-miR-650	AL021707.2	
hsa-miR-650	AL031595.3	
hsa-miR-650	Z95331.1	
hsa-miR-650	AL121672.2	
hsa-miR-650	FP325332.1	
hsa-miR-650	Z84468.1	
hsa-miR-650	C22orf34	
hsa-miR-650	CHKB-AS1	
hsa-miR-650	AC004656.1	
hsa-miR-650	AC092198.1	
hsa-miR-650	AC233728.1	
hsa-miR-650	JPX	
hsa-miR-650	FTX	
hsa-miR-650	AL359740.1	
hsa-miR-650	AL035425.4	
hsa-miR-650	AL645820.1	
hsa-miR-650	MIR503HG	
hsa-miR-650	LINC00894	
hsa-miR-650	TTTY10	
hsa-miR-760	LINC01128	
hsa-miR-760	AL645608.3	
hsa-miR-760	AL645608.1	
hsa-miR-760	AL645608.8	
hsa-miR-760	AL391244.1	
hsa-miR-760	AL031847.1	
hsa-miR-760	LINC01672	
hsa-miR-760	LINC01759	
hsa-miR-760	PIK3CD-AS2	
hsa-miR-760	AL450998.2	
hsa-miR-760	AL137798.2	
hsa-miR-760	AL021920.3	
hsa-miR-760	BX284668.5	
hsa-miR-760	ZNF436-AS1	
hsa-miR-760	AL591845.1	
hsa-miR-760	AL050341.2	
hsa-miR-760	SLFNL1-AS1	
hsa-miR-760	AC096540.1	
hsa-miR-760	AL512353.1	
hsa-miR-760	ERI3-IT1	
hsa-miR-760	LINC01144	
hsa-miR-760	FOXD2-AS1	
hsa-miR-760	LINC01748	
hsa-miR-760	AL359504.2	
hsa-miR-760	MIR137HG	
hsa-miR-760	AC093157.1	
hsa-miR-760	AL359258.1	
hsa-miR-760	AL390038.1	
hsa-miR-760	AL160006.1	
hsa-miR-760	AL365361.1	
hsa-miR-760	AL390066.1	
hsa-miR-760	RP6-42F4.1	
hsa-miR-760	LINC01719	
hsa-miR-760	AC244394.2	
hsa-miR-760	AL365436.2	
hsa-miR-760	RUSC1-AS1	
hsa-miR-760	AL355388.2	
hsa-miR-760	AL365181.3	
hsa-miR-760	AL590560.2	
hsa-miR-760	AL451074.2	
hsa-miR-760	AL513217.1	
hsa-miR-760	MIR29B2CHG	
hsa-miR-760	AL359551.1	
hsa-miR-760	AL591848.3	
hsa-miR-760	LINC01341	
hsa-miR-760	LINC01814	
hsa-miR-760	AC007249.2	

hsa-miR-760	AC138655.1	
hsa-miR-760	AC007744.1	
hsa-miR-760	PCBP1-AS1	
hsa-miR-760	LINC01102	
hsa-miR-760	LINC01918	
hsa-miR-760	MIR4435-2HG	
hsa-miR-760	AC114763.1	
hsa-miR-760	HAGLR	
hsa-miR-760	SATB2-AS1	
hsa-miR-760	LINC01963	
hsa-miR-760	AC098820.1	
hsa-miR-760	LINC01237	
hsa-miR-760	AC022007.1	
hsa-miR-760	GHRLOS	
hsa-miR-760	SH3BP5-AS1	
hsa-miR-760	AC090948.2	
hsa-miR-760	AC132807.1	
hsa-miR-760	LINC01967	
hsa-miR-760	ITGA9-AS1	
hsa-miR-760	ACVR2B-AS1	
hsa-miR-760	AC099329.2	
hsa-miR-760	AC006058.1	
hsa-miR-760	KIF9-AS1	
hsa-miR-760	AC104447.1	
hsa-miR-760	U73166.1	
hsa-miR-760	AC115284.1	
hsa-miR-760	AC096887.2	
hsa-miR-760	PSMD6-AS2	
hsa-miR-760	DUBR	
hsa-miR-760	AC022336.3	
hsa-miR-760	AC084035.1	
hsa-miR-760	AFAP1-AS1	
hsa-miR-760	AC097381.1	
hsa-miR-760	UBA6-AS1	
hsa-miR-760	FAM198B-AS1	
hsa-miR-760	EXOC3-AS1	
hsa-miR-760	SLC9A3-AS1	
hsa-miR-760	AC025171.2	
hsa-miR-760	AC025171.1	
hsa-miR-760	VCAN-AS1	
hsa-miR-760	AC020923.1	
hsa-miR-760	LUCAT1	
hsa-miR-760	AC008840.1	
hsa-miR-760	AC008467.1	
hsa-miR-760	EPB41L4A-AS1	
hsa-miR-760	LINC01184	
hsa-miR-760	MIR3936HG	
hsa-miR-760	C5orf66-AS2	
hsa-miR-760	C5orf66	
hsa-miR-760	AC012613.2	
hsa-miR-760	CARMN	
hsa-miR-760	AC008429.1	
hsa-miR-760	AC145098.2	
hsa-miR-760	AC008443.1	
hsa-miR-760	LINC01011	
hsa-miR-760	AL391422.3	
hsa-miR-760	AL354740.1	
hsa-miR-760	Z97832.2	
hsa-miR-760	AL592158.1	
hsa-miR-760	AL359711.2	
hsa-miR-760	AL356234.2	
hsa-miR-760	AGPAT4-IT1	
hsa-miR-760	PACRG-AS1	
hsa-miR-760	AC073957.2	
hsa-miR-760	AC004540.1	
hsa-miR-760	AC004080.6	
hsa-miR-760	AC007285.1	
hsa-miR-760	LINC01176	
hsa-miR-760	AC005154.1	
hsa-miR-760	POLR2J4	
hsa-miR-760	LINC00174	
hsa-miR-760	AC006330.1	
hsa-miR-760	STAG3L5P-PVRIG2P-PILRB	

hsa-miR-760	AC004014.2	
hsa-miR-760	AC019155.2	
hsa-miR-760	SND1-IT1	
hsa-miR-760	PRKAG2-AS1	
hsa-miR-760	AC006017.1	
hsa-miR-760	PAXIP1-AS1	
hsa-miR-760	AC144652.1	
hsa-miR-760	AC021218.1	
hsa-miR-760	AC100810.1	
hsa-miR-760	AC027117.1	
hsa-miR-760	AC090197.1	
hsa-miR-760	MIR124-2HG	
hsa-miR-760	LINC01111	
hsa-miR-760	AC009686.2	
hsa-miR-760	CA3-AS1	
hsa-miR-760	OTUD6B-AS1	
hsa-miR-760	GASAL1	
hsa-miR-760	PRNCR1	
hsa-miR-760	PVT1	
hsa-miR-760	ASAP1-IT2	
hsa-miR-760	MINCR	
hsa-miR-760	AC105118.1	
hsa-miR-760	AC067930.6	
hsa-miR-760	AC084125.2	
hsa-miR-760	AL606807.1	
hsa-miR-760	AL807761.4	
hsa-miR-760	AL441992.1	
hsa-miR-760	LINC01503	
hsa-miR-760	LINC00963	
hsa-miR-760	AL360004.1	
hsa-miR-760	AL358781.1	
hsa-miR-760	SNHG7	
hsa-miR-760	ARRDC1-AS1	
hsa-miR-760	AL139125.2	
hsa-miR-760	GATA3-AS1	
hsa-miR-760	AL157392.3	
hsa-miR-760	C10orf25	
hsa-miR-760	AC022532.1	
hsa-miR-760	AC022400.7	
hsa-miR-760	ZSWIM8-AS1	
hsa-miR-760	AL132656.4	
hsa-miR-760	NUTM2B-AS1	
hsa-miR-760	LINC00858	
hsa-miR-760	AL138921.1	
hsa-miR-760	AL162274.3	
hsa-miR-760	LINC01165	
hsa-miR-760	AC137894.1	
hsa-miR-760	AP006284.1	
hsa-miR-760	H19	
hsa-miR-760	IGF2-AS	
hsa-miR-760	KCNQ1OT1	
hsa-miR-760	LINC00958	
hsa-miR-760	BBOX1-AS1	
hsa-miR-760	SNHG1	
hsa-miR-760	AP001453.1	
hsa-miR-760	NEAT1	
hsa-miR-760	MALAT1	
hsa-miR-760	AP001266.1	
hsa-miR-760	AP001157.1	
hsa-miR-760	AP000785.1	
hsa-miR-760	AP000757.1	
hsa-miR-760	MIR100HG	
hsa-miR-760	AC125807.2	
hsa-miR-760	FAM66C	
hsa-miR-760	AC092821.3	
hsa-miR-760	AC009509.1	
hsa-miR-760	AC009318.4	
hsa-miR-760	AC068987.4	
hsa-miR-760	AC025259.1	
hsa-miR-760	AC068888.1	
hsa-miR-760	AC073573.1	
hsa-miR-760	HOXC-AS2	
hsa-miR-760	AC025165.1	

hsa-miR-760	AC020656.2	
hsa-miR-760	LINC02444	
hsa-miR-760	AC121761.2	
hsa-miR-760	LINC02407	
hsa-miR-760	TMPO-AS1	
hsa-miR-760	HELLPAR	
hsa-miR-760	AC007541.1	
hsa-miR-760	PXN-AS1	
hsa-miR-760	AC069234.3	
hsa-miR-760	LINC01089	
hsa-miR-760	AC027290.2	
hsa-miR-760	AC026362.1	
hsa-miR-760	AC026362.2	
hsa-miR-760	AC117503.3	
hsa-miR-760	AC122688.3	
hsa-miR-760	FZD10-AS1	
hsa-miR-760	LINC01257	
hsa-miR-760	AC131009.3	
hsa-miR-760	AC131212.3	
hsa-miR-760	AC131212.4	
hsa-miR-760	N4BP2L2-IT2	
hsa-miR-760	DLEU1	
hsa-miR-760	RBM26-AS1	
hsa-miR-760	MIR17HG	
hsa-miR-760	LINC00551	
hsa-miR-760	SOX1-OT	
hsa-miR-760	AL442125.1	
hsa-miR-760	AL096870.2	
hsa-miR-760	G2E3-AS1	
hsa-miR-760	LINC01588	
hsa-miR-760	FRMD6-AS1	
hsa-miR-760	FUT8-AS1	
hsa-miR-760	AC005519.1	
hsa-miR-760	AL049780.1	
hsa-miR-760	AF111167.2	
hsa-miR-760	VASH1-AS1	
hsa-miR-760	LINC01550	
hsa-miR-760	MEG3	
hsa-miR-760	MEG9	
hsa-miR-760	DIO3OS	
hsa-miR-760	LINC02323	
hsa-miR-760	SNHG14	
hsa-miR-760	ARHGAP11B	
hsa-miR-760	AC037198.1	
hsa-miR-760	AC020661.1	
hsa-miR-760	AC013564.1	
hsa-miR-760	AC100827.3	
hsa-miR-760	LOXL1-AS1	
hsa-miR-760	AC091230.1	
hsa-miR-760	SNHG21	
hsa-miR-760	LINC01586	
hsa-miR-760	MIR9-3HG	
hsa-miR-760	AC087284.1	
hsa-miR-760	ZNF710-AS1	
hsa-miR-760	IDH2-DT	
hsa-miR-760	LINC00923	
hsa-miR-760	AL008727.1	
hsa-miR-760	AL133297.2	
hsa-miR-760	AL031009.1	
hsa-miR-760	SNHG9	
hsa-miR-760	AC106820.2	
hsa-miR-760	AC106820.4	
hsa-miR-760	AC093525.8	
hsa-miR-760	AC093525.9	
hsa-miR-760	MMP25-AS1	
hsa-miR-760	LCMT1-AS2	
hsa-miR-760	AC008741.2	
hsa-miR-760	IL21R-AS1	
hsa-miR-760	AC002551.1	
hsa-miR-760	AC145285.6	
hsa-miR-760	AC009133.21	
hsa-miR-760	AC135050.6	
hsa-miR-760	AC026471.6	

hsa-miR-760	AC092368.3	
hsa-miR-760	AC106779.1	
hsa-miR-760	AC009090.5	
hsa-miR-760	AC092378.1	
hsa-miR-760	AC018557.1	
hsa-miR-760	AC040160.2	
hsa-miR-760	AC040160.1	
hsa-miR-760	AC027682.2	
hsa-miR-760	AC027682.3	
hsa-miR-760	AC009022.1	
hsa-miR-760	LINC01572	
hsa-miR-760	AC140912.1	
hsa-miR-760	AC009078.3	
hsa-miR-760	AC092718.2	
hsa-miR-760	AC092139.3	
hsa-miR-760	ATP2C2-AS1	
hsa-miR-760	LINC02188	
hsa-miR-760	AC010531.4	
hsa-miR-760	AC105429.1	
hsa-miR-760	AC010536.1	
hsa-miR-760	AC126696.2	
hsa-miR-760	SNAI3-AS1	
hsa-miR-760	LINC00304	
hsa-miR-760	AC087392.3	
hsa-miR-760	AC099684.2	
hsa-miR-760	AC006435.2	
hsa-miR-760	AC015799.1	
hsa-miR-760	AC005696.4	
hsa-miR-760	AC012146.1	
hsa-miR-760	AC016876.2	
hsa-miR-760	AC118755.2	
hsa-miR-760	LRRC75A-AS1	
hsa-miR-760	AC124066.1	
hsa-miR-760	AC010761.1	
hsa-miR-760	AC127024.2	
hsa-miR-760	AC015849.5	
hsa-miR-760	AC009283.1	
hsa-miR-760	AC067852.3	
hsa-miR-760	RAMP2-AS1	
hsa-miR-760	ASB16-AS1	
hsa-miR-760	AC142472.1	
hsa-miR-760	AC008105.3	
hsa-miR-760	AC008105.1	
hsa-miR-760	AC018521.1	
hsa-miR-760	TBX2-AS1	
hsa-miR-760	AC005828.5	
hsa-miR-760	ARHGAP27P1-BPTFP1-KPNA2P3	
hsa-miR-760	AC097641.2	
hsa-miR-760	AC022211.2	
hsa-miR-760	TEN1-CDK3	
hsa-miR-760	SNHG16	
hsa-miR-760	AC111170.2	
hsa-miR-760	LINC01993	
hsa-miR-760	AC087645.2	
hsa-miR-760	AC087741.1	
hsa-miR-760	AC120024.1	
hsa-miR-760	BAIAP2-AS1	
hsa-miR-760	LINC00482	
hsa-miR-760	AC110285.2	
hsa-miR-760	AC139530.1	
hsa-miR-760	LINC01895	
hsa-miR-760	DLGAP1-AS1	
hsa-miR-760	AP005671.1	
hsa-miR-760	NDUFV2-AS1	
hsa-miR-760	AP001269.4	
hsa-miR-760	AP005131.6	
hsa-miR-760	AC090206.1	
hsa-miR-760	AC090220.1	
hsa-miR-760	AC021504.1	
hsa-miR-760	AC090340.1	
hsa-miR-760	AC105105.2	
hsa-miR-760	AC105105.1	
hsa-miR-760	AC009716.2	

hsa-miR-760	AC018413.1	
hsa-miR-760	AC093330.2	
hsa-miR-760	AC139100.2	
hsa-miR-760	AC009005.1	
hsa-miR-760	AC005329.1	
hsa-miR-760	AC027307.3	
hsa-miR-760	AC027307.2	
hsa-miR-760	AC005306.1	
hsa-miR-760	AC092068.3	
hsa-miR-760	AC011498.7	
hsa-miR-760	AC104532.2	
hsa-miR-760	ZNF561-AS1	
hsa-miR-760	AC008764.7	
hsa-miR-760	AC008764.10	
hsa-miR-760	AC011447.7	
hsa-miR-760	LINC01224	
hsa-miR-760	AC007786.3	
hsa-miR-760	LINC01782	
hsa-miR-760	AC008738.7	
hsa-miR-760	AC010485.1	
hsa-miR-760	AC020910.4	
hsa-miR-760	TMEM147-AS1	
hsa-miR-760	AD001527.2	
hsa-miR-760	LINC00665	
hsa-miR-760	LINC01535	
hsa-miR-760	AC011479.3	
hsa-miR-760	LIPE-AS1	
hsa-miR-760	AC243964.3	
hsa-miR-760	IGFL2-AS1	
hsa-miR-760	AC093503.1	
hsa-miR-760	AC008895.1	
hsa-miR-760	AC073548.1	
hsa-miR-760	AC010331.1	
hsa-miR-760	AC011450.1	
hsa-miR-760	PTOV1-AS1	
hsa-miR-760	AC010327.4	
hsa-miR-760	AC010642.2	
hsa-miR-760	AC012313.3	
hsa-miR-760	A1BG-AS1	
hsa-miR-760	LINC00261	
hsa-miR-760	AL117381.1	
hsa-miR-760	NORAD	
hsa-miR-760	AL031666.2	
hsa-miR-760	CEBPB-AS1	
hsa-miR-760	AL109930.1	
hsa-miR-760	APCDD1L-AS1	
hsa-miR-760	MIR646HG	
hsa-miR-760	AL121832.3	
hsa-miR-760	LINC00659	
hsa-miR-760	MIR548XHG	
hsa-miR-760	AP000695.3	
hsa-miR-760	TRPM2-AS	
hsa-miR-760	LINC01547	
hsa-miR-760	LINC00205	
hsa-miR-760	LINC01694	
hsa-miR-760	AJ239328.1	
hsa-miR-760	MCM3AP-AS1	
hsa-miR-760	AC016026.1	
hsa-miR-760	LINC01637	
hsa-miR-760	AC002470.2	
hsa-miR-760	THAP7-AS1	
hsa-miR-760	GUSBP11	
hsa-miR-760	AP000347.2	
hsa-miR-760	AP000350.6	
hsa-miR-760	AC253536.3	
hsa-miR-760	ADORA2A-AS1	
hsa-miR-760	AL008721.2	
hsa-miR-760	MIAT	
hsa-miR-760	TTC28-AS1	
hsa-miR-760	TUG1	
hsa-miR-760	AC005005.4	
hsa-miR-760	FP325335.1	
hsa-miR-760	AL021707.1	

hsa-miR-760	AL031595.3	
hsa-miR-760	AL079301.1	
hsa-miR-760	Z82243.1	
hsa-miR-760	Z82186.1	
hsa-miR-760	AL117329.1	
hsa-miR-760	FP325330.3	
hsa-miR-760	BX890604.1	
hsa-miR-760	FAM239B	
