# Supplementary material for: Effect of aberrant fructose metabolism following SARS-CoV-2 infection on colorectal cancer patients’ poor prognosis
Source: PLoS Comput Biol. 2024 Sep 27;20(9):e1012412. doi: 10.1371/journal.pcbi.1012412 (PMC11463760; doi:10.1371/journal.pcbi.1012412)
Supplement: Attachment — Submitted filename: Response to Reviewers.docx [file pcbi.1012412.s007.docx]

Dear Editors and Reviewers,

Thank you very much for your letter and for the reviewers' valuable and helpful comments concerning our manuscript (ID: PCOMPBIOL-D-23-00881). We have studied the comments carefully and made a major revision in accordance with your comments. The revised parts have been marked in the “Tracked Changes” in the revised manuscript. We hope that this revised manuscript will meet the high-quality standard of you. The responds to the reviewers’ comments are as following:

**Reviewer #1:**

SARS-CoV-2-induced cancer patients have a poor prognosis, which may be related to fructose metabolism. In order to improve clinical practice, a composite predictive nomogram is presented in this work. It includes characteristics of the aberrant fructose metabolism brought on by this novel coronavirus, together with age and tumor stage. The genes with the best possible prognostic values were found using LASSO regression analysis. This work lays the theoretical foundation for future customized treatment of fructose metabolism in these patients, while also optimizing dietary advice and therapeutic care for patients with colorectal cancer in the context of the COVID-19 pandemic.

The manuscript is well written. Furthermore, there are other issues that need to be clarified in a revised version.

More detailed comments are provided below.

**1. The images are of very poor quality, can’t even understandable. Please re-upload all the images with proper legends and titles.**

**Response:** I'm sorry for the inconvenience. It was verified that the quality of the uploaded pictures met the requirements of the journal, and the unclear pictures may be because the pictures were compressed when sent to the reviewers. As suggested, we re-uploaded high-quality images with proper legends and titles in the revised version. And in the integrated PDF file, there is an original image download link located in the top right corner of each figure.

**2. For 1B image, consider the cut-off in log2(FC) so that selected number of genes can be identified properly.**

**Response:** I am sorry that this step has brought you controversial misunderstandings. Screening conditions for differential genes were adj.p < 0.05. In differential gene screening, differentially expressed genes are usually identified according to certain screening conditions. Among them, the adjusted P value (adj) is used to evaluate whether the difference reaches a statistically significant level, which can help us control the false alarm rate of multiple hypothesis testing. Typically, logFC is set to screen for genes with large differential expression. However, setting the logFC to 0 means that differences in expression are not considered and are screened as differentially expressed genes as long as there are differences. In the subsequent analysis, we will use other algorithms to select the most representative signature genes, so in this step of analysis we include as many differential genes as possible.

**3. Accuracy can not be observed properly as the ROC curve is not clear to view. So that the results can be verified.**

**Response:** We apologize for the resolution of the images. After verification, it was found that the pictures we uploaded were clear and met all the requirements of the journal, and the reason for the blurred pictures may be because the pictures were compressed when the magazine forwarded the reviewers.

**4. Here, authors mentioned that they have splitted the dataset into 4:1 ratio for training and validation purpose randomly. Please verify that this ratio performing best or not.**

**Response:** Your review and insightful comments regarding our study are greatly appreciated. We regret that we were unable to furnish a precise substantiation of the ratio between the training set and testing set that you referenced for machine learning. Although we adhered to the 4:1 ratio that is widely recognized in the academic community, we neglected to conduct validation using this ratio. By assimilating patterns and rules from training set samples, machine learning models acquire the ability to generate predictions regarding unobserved data. The training set generally comprises a substantial quantity of samples and encompasses a wide range of scenarios that the model might confront during the implementation stage. After the model learns on the training set, it needs to be evaluated on the test set to understand its performance on unseen data. The test set should be independent of the training set, ensuring that the model has not seen these data in advance. Typically 80% of the dataset is used as the training set and 20% as the test set. The training set and test set should be divided before building the model to avoid data peeping errors, that is, to prevent the model from performing too optimistically on the test set and the actual performance is not as expected due to too much understanding of the sample characteristics in the test set. This division is to ensure that the training set has sufficient data to train the model, while the test set is also large enough to provide an effective assessment of the model performance. And the training set and the test set can represent the characteristics of the whole data set. We acknowledge that the interpretability and dependability of our findings may be impacted as a result. In subsequent endeavors, we shall endeavor to validate and investigate this crucial matter with greater thoroughness so as to bolster the integrity and openness of our research. Once more, I appreciate your review and insightful feedback.

**5.Here Lasso regression technique has been implemented. It would be great to compare this regression model with some other techniques? please add one comparison table.**

**Response:** Thank you very much for your valuable comments and suggestions. We value your interest and guidance in our research. We apologize for failing to provide a comparison table of the regression models and other techniques you mentioned. Because our study focused on other aspects, we did not make a direct comparison of these techniques. Our aim is to explore new ideas and approaches in specific areas rather than to compare them with existing technologies. However, we welcome and encourage future studies to explore and compare more deeply in this regard. This will help to enhance our understanding of methods and technologies and to advance the field. Thank you again for your review and valuable suggestions. Any additional comments you make will be carefully considered and adjusted accordingly in the revised manuscript. Second, our prognostic model is an innovative and prospective prognostic model because we constructed a prognostic model in CRC based on differential fructose metabolism genes in patients with novel coronavirus, breaking the category that traditional prognostic models consider only a single disease. This innovative perspective can provide a more comprehensive and comprehensive prognostic evaluation. Fructose metabolism may play an important role in tumor development. By investigating the role of fructose metabolism genes, the biological mechanism of colorectal cancer can be better understood, and the accuracy and validity of prognostic models can be provided with higher reliability.

**Reviewer #2:**

This study aims to use a bioinformatics approach to predict fructose metabolism genes that are altered in response to COVID-19 infection and also in tissues from CRC patients, and are linked to CRC prognosis. They developed a prognostic risk score based on a number of these genes, and used this to examine correlations to immune responses and CRC treatment efficacy. The aims of the research have been performed well, there are multiple areas in which the rationale could be explained clearer. The discussion does not discuss any of the results generated in the paper. Therefore, the significance of their findings is not clear, or what their findings will lead to, or how they will be followed up. The outcome of the research is observational, but an interesting hypothesis has been generated.

Major Comments: In most cases, the rationale for performing each type of analysis is not mentioned, there is also no summary of findings in each section which would help link the results to the next analysis. Also, the discussion could be reorganized to discuss the results of the study, it is currently a review of fructose metabolism. There is no mention of any of the findings, or limitations of their work.

**Response:** Your guidance strongly endorses the importance of reorganizing the discussion to better present the study results and highlight the findings. We will focus on the findings and ensure a deeper discussion of fructose metabolism to present our findings. In addition, we will ensure that the limitations of our work are mentioned in the discussion to facilitate a comprehensive understanding of the study.

Minor Comments:

**1. Language in abstract needs improvement**

**Response:** As suggested, we carefully organized and edited these sections in the revised version.

**2. The statement “SARS-CoV-2 is more prevalent among cancer patients treated with anticancer therapy than those without anticancer therapy” as a supporting argument needs more thought as this could be due to their increased exposure when not distancing vs anything biological.**

**Response:** We highly value your insightful remarks and wholeheartedly concur with your viewpoint. We failed to account for the possibility that the correlation between anti-cancer therapy and the incidence of SARS-CoV-2 infection might be affected by additional variables, such as an elevated risk of organism contact, in our study. Taking this perspective into consideration, we shall delve deeper into this matter. We would like to extend our sincere appreciation to the reviewers for their invaluable guidance and suggestions. Your insights have significantly improved our study, and we will duly incorporate and modify them in the revised manuscript. We have incorporated the additional content as suggested into the 78 to 79 line of the revised manuscript.

**3. The supporting citations for the statement the fructose can make cancer worse are for breast and prostate cancers, not CRC. As metabolism differs even by stage and genetic mutations in CRC it is difficult to translate the findings in other cancers to CRC. So this statement should express that limitation.**

**Response:** We greatly appreciate your review and insightful feedback. We have incorporated and elaborated on the research progress you discussed regarding the cancer-promoting impact of fructose on colorectal cancer in the paper's discussion section. We elaborate on this topic in the discussion and cite the most recent research findings to support our views. A study in mice showed that when fructose doses exceed 1 g/kg, fructose absorption in the small intestine is weakened, resulting in increased fructose concentrations in the large intestine. Moreover, fructose can be captured by tumors in the intestinal lumen rather than being delivered to the liver and blood. We have incorporated the additional content as suggested into the 368 to 375 line of the revised manuscript. Your guidance is extraordinarily beneficial to our research. And we consistently striving to enhance the paper will guarantee its credibility and quality.

**4. Information on the sex, age, stage and types of tissues analyzed and method of analysis (for example GSE41258 is microarray) in the 3 CRC datasets would be helpful, perhaps as a supplementary table. The lung covid dataset (GSE183533) as generated by RNA-Seq, perhaps this could be mentioned, it’s possible some transcripts are missed in the microarray?**

**Response:** We have organized your feedback into a table to upload as supplementary material to a file named Supplementary Table S5.

**5. Fig 2, It is not clear why the OS time is different between each plot on Figs 2. Typically this is set to 5 years, or in some cases 10 years for CRC, which is the timeframe in which recurrence may occur. This should be more clinically relevant.**

**Response:** We greatly appreciate your review and insightful feedback. With respect to the external datasets that were utilized to verify the model's robustness, you made reference to the inconsistent OS that were present. We regret the omission of this information in the paper and appreciate your drawing attention to it. In order to enhance the demonstration of the prognostic model's robustness, the external dataset was chosen at random, and the consistency of the participants' overall survival was not controlled for. Subsequent investigations will incorporate a more stringent evaluation process for external datasets in order to ascertain the model's dependability and suitability.

**6. The rationale for examining immune infiltration was not explained, is this linked to fructose metabolism?**

**Response:** I express my gratitude for your evaluation and direction. The rationales for investigating immune infiltrates have been appended to the appropriate sections of the manuscript and the pertinent material has been expanded. Recent findings suggest that tumor-infiltrating immune cells' characteristics are increasingly linked to the onset and advancement of cancer It is a remarkable fact that dietary stress exacerbates the metabolic tug of war in tumors and directly affects local CD8+ T cell function. We have incorporated the additional content as suggested into the 228 to 237 line of the revised manuscript. Your recommendations are extremely beneficial to our research, and we will continue to work diligently to ensure the paper's accuracy and quality. Should you have any further remarks or recommendations, we would be delighted to receive and duly consider them.

**7. Fig 3. A – the risk groups should be labelled in the legend. B, it is not clear which 2 groups are being compared, are these high and low risk groups again?**

**Response:** As the reviewer concerned, legends have been appended to the figures in the paper in order to augment the readers' comprehension and interpretation of the visual material. Moreover, Fig. 3. B represents a correlation analysis, which examines the correlation between immune factors across all patients. Since it does not involve a comparison between two distinct groups, it does not distinguish between high-risk and low-risk individuals; rather, it comprises a sample of both the high-risk and low-risk groups. The objective of correlation analysis among immune factors is frequently to gain a comprehensive comprehension of the immune system as a whole, as opposed to focusing on particular diseases or risk factors. Therefore, pooling all samples for analysis allows a more comprehensive understanding of the relationship between immune factors without being restricted to specific risk groups. Furthermore, the immune system is an exceedingly intricate system comprised of numerous interdependent components. As a result, dividing immune factor analyses into components associated with high and low risk may oversimplify the issue and fail to capture the complexity of the immune system as a whole. It is crucial to note that immune factor correlation analysis frequently necessitates significant data support in order to guarantee the results' dependability and statistical significance. Excessive group analysis may result in inadequate sample size for each group, thereby compromising the dependability of the analysis findings. Hence, it is customary and more suitable to conduct immune factor correlation analysis on the entirety of the samples so as to gain a more comprehensive understanding of the immune system's intricacy. This determination has been reached after a thorough evaluation of our comprehension and methodology regarding the research inquiry. Should you have any further remarks or recommendations, we would be delighted to engage in further discussion. Again, I appreciate your assistance and professional guidance.

**8. Gender should be changed to sex in text and on figures.**

**Response:** As the reviewer correctly pointed out, in the paper text and figures, we have replaced "gender" with "sex" to more precisely represent the pertinent variables in our research.

**9. Fig 4, what was the rationale for performing univariate and multivariate analysis.**

**Response:** Univariate multivariate analysis was performed using the survival package to fit risk scores and clinical symptoms according to the coxph function. First, we could fit a Cox proportional-hazards model, a survival analysis model for assessing time-related data, using the coxph function. We can use this function to evaluate the independent impact of clinical symptoms and risk score on the prognosis of colorectal cancer patients by fitting the model with clinical symptoms and risk score as independent variables and survival time and survival status of patients as dependent variables, and we can obtain the relative hazard ratio (HR) of each variable as well as the associated confidence interval and significance level.

**10. There is no information provided on the antibodies used for immunohistochemistry, or their authentication.**

**Response:** We included the information in the Materials and Methods section in the revised version. Formalin-fixed paraffin-embedded sample specimens of patients with colorectal cancer were obtained from the Human Protein Atlas Project(https://www.proteinatlas.org). As part of the sample collection supervised by the Uppsala Biobank (http://www.uppsalabiobank.uu.se/en), cancer tissues utilized for protein expression analysis were collected from the Department of Pathology at Uppsala University Hospital, Uppsala, Sweden.
