# Supplementary material for: Effect of aberrant fructose metabolism following SARS-CoV-2 infection on colorectal cancer patients’ poor prognosis
Source: PLoS Comput Biol. 2024 Sep 27;20(9):e1012412. doi: 10.1371/journal.pcbi.1012412 (PMC11463760; doi:10.1371/journal.pcbi.1012412)
Supplement: Attachment — Submitted filename: To Reviewer.docx [file pcbi.1012412.s008.docx]

Dear Editors and Reviewers,

Thank you very much for your letter and for the reviewers' valuable and helpful comments concerning our manuscript (ID: PCOMPBIOL-D-23-00881). We have studied the comments carefully and made a minor revision in accordance with your comments. The revised parts have been marked in the “Tracked Changes” in the revised manuscript. We hope that this revised manuscript will meet the high-quality standard of you. The responds to the reviewers’ comments are as following:

**Reviewer #1:**

The authors have addressed my previous comments satisfactorily.

**1. The authors conducted various computational analyses for this study; however, the script for these analyses is not included in the manuscript.**

**Response:** I hope this message finds you well. I am writing to inform you that we have uploaded supplementary materials, including the relevant code used in our manuscript, as requested. These materials should assist in the evaluation of our research. Regarding the code, we have included most of it in the supplementary files for your review. We have provided as much detail as possible in the supplementary materials to facilitate transparency and reproducibility. We believe that the provided materials are comprehensive and will facilitate a thorough assessment of our findings. Thank you for your attention to this matter. Please let us know if you require any further information or assistance.

**2. The authors have included links to various databases throughout the manuscript. Instead of providing these links, please cite the original papers associated with each database.**

**Response:** I hope this message finds you well. I wanted to inform you that we have now appropriately referenced the original papers related to the datasets used in our manuscript. We believe that these references will provide clarity and ensure proper acknowledgment of the original sources of the datasets employed in our study. Thank you for your attention to this matter. Please feel free to reach out if you have any further questions or require additional information.

**Reviewer #2:**

The authors have been responsive to the recommendations made, however the discussion still mostly discusses research from other studies, and not the results of their own study. The discussion is also very long and given that the research from the paper is not really discussed in any detail, there is room for improvement here.

**Response:** I hope this message finds you well. I am writing to inform you that we have now added a comprehensive discussion of our study in the manuscript's Discussion section. This addition aims to provide a thorough analysis and interpretation of our findings in the context of existing literature and implications for the field. We believe that this detailed discussion will address the points raised during the review process and enhance the clarity and depth of our manuscript. Thank you for your consideration of this update. Please let us know if you require any further clarification or have additional feedback.
